# miR-155 Controls Lymphoproliferation in LAT Mutant Mice by Restraining T-Cell Apoptosis via SHIP-1/mTOR and PAK1/FOXO3/BIM Pathways

**DOI:** 10.1371/journal.pone.0131823

**Published:** 2015-06-29

**Authors:** Alexandre K. Rouquette-Jazdanian, Robert L. Kortum, Wenmei Li, Robert K. Merrill, Phan H. Nguyen, Lawrence E. Samelson, Connie L. Sommers

**Affiliations:** Laboratory of Cellular and Molecular Biology, Center for Cancer Research, National Cancer Institute, National Institutes of Health, Bethesda, Maryland, United States of America; School of Medicine, University of Belgrade, SERBIA

## Abstract

Linker for Activation of T cells (LAT) is an adapter protein that is essential for T cell function. Knock-in mice with a LAT mutation impairing calcium flux develop a fatal CD4^+^ lymphoproliferative disease. miR-155 is a microRNA that is correlated with hyperproliferation in a number of cancers including lymphomas and leukemias and is overexpressed in mutant LAT T cells. To test whether miR-155 was merely indicative of T cell activation or whether it contributes to lymphoproliferative disease in mutant LAT mice, we interbred LAT mutant and miR-155-deficient mice. miR-155 deficiency markedly inhibited lymphoproliferative disease by stimulating BIM-dependent CD4^+^ T cell apoptosis, even though ERK activation and T cell proliferation were increased in double mutant CD4^+^ T cells. Bim/*Bcl2l11* expression is activated by the forkhead transcription factor FOXO3. Using miR-155-deficient, LAT mutant T cells as a discovery tool, we found two connected pathways that impact the nuclear translocation and activation of FOXO3 in T cells. One pathway is mediated by the inositide phosphatase SHIP-1 and the serine/threonine kinases AKT and PDK1. The other pathway involves PAK1 and JNK kinase activation. We define crosstalk between the two pathways via the kinase mTOR, which stabilizes PAK1. This study establishes a role for PAK1 in T cell apoptosis, which contrasts to its previously identified role in T cell proliferation. Furthermore, miR-155 regulates the delicate balance between PAK1-mediated proliferation and apoptosis in T cells impacting lymphoid organ size and function.

## Introduction

Engagement of the T cell antigen receptor (TCR) initiates a number of downstream signaling pathways resulting in T cell activation and proliferation. These activation events enable helper (CD4^+^) T cells to produce cytokines and activate other immune cells resulting in a functional adaptive immune response. One immediate result of TCR activation is the tyrosine phosphorylation of many signaling proteins in CD4^+^ T cells. A protein that is heavily phosphorylated on tyrosine following TCR stimulation is the adapter protein LAT (Linker for activation of T cells). Upon TCR stimulation, LAT is phosphorylated on multiple tyrosines, one of which (Y132 in human LAT, Y136 in mouse LAT) then acts as a docking site for the phospholipase PLC-γ1. Other LAT tyrosines act as docking sites for numerous other signaling molecules (see[1]). Previously we and others generated knock-in mice that express LAT with Y136 mutated to F136, hereafter referred to as LAT-KI mice [2, 3]. These mice have an early block in T cell maturation but later develop a lymphoproliferative disease characterized by marked splenomegaly and lymphadenopathy. The disease is dependent on LAT mutant T cells [4] and the mice show a hyper-proliferation of CD4^+^ helper T cells that secrete large amounts of the signature Th2 cytokine, IL-4 [2, 3].

We have been interested in determining what known and novel pathways of T cell signaling drive LAT-KI T cell proliferation. In classical TCR signaling pathways in normal T cells, TCR activation results in protein tyrosine kinase activation, LAT phosphorylation, and PLC-γ1 recruitment and activation. PLC-γ1 converts PIP3 to IP3 and DAG. Increases in IP3 levels lead to calcium influx. Increases in DAG levels lead to activation of PKCs and RASGRP, a RasGEF that activates RAS, which leads to the activation of ERK [1]. In LAT-KI peripheral T cells, TCR-induced calcium influx was absent, as predicted from the loss of PLC-γ1 activity. Unexpectedly, ERK activation was observed in LAT-KI T cells [5].

We have used LAT-KI mice as a discovery tool to define T cell signaling pathways and their functions. We have recently published two studies that address how ERK can be activated in LAT-KI T cells. LAT-KI lymphoproliferative disease is largely dependent on RASGRP. In the first study, we addressed how RASGRP could be activated in LAT-KI T cells in the absence of PLC-γ1-dependent DAG production [6]. In LAT-KI T cells, hyperactive LCK associates with and activates PKCθ, which then phosphorylates and activates RASGRP1. This alternative pathway operates in the absence of classical PLC-γ1 activation in LAT-KI T cells. The second study described another mechanism for ERK activation that is operational in both wild-type and LAT-KI T cells. This pathway involves signaling through a trimolecular complex composed of the serine/threonine kinase PAK1, the adapter protein BAM32, and PLC-γ1, in which PLC-γ1 acts as a scaffold and not as an enzyme [7]. In this complex, PLC-γ1 binding dissociates PAK1 inhibitory homodimers rendering PAK1 active. Active PAK1 can phosphorylate the serine/threonine kinases RAF and MEK, thereby activating ERK and JNK. This pathway is engaged in LAT-KI T cells because it utilizes non-catalytic (non-phosphorylated) PLC-γ1 and is LAT-independent. However the pathway is also operational in wild-type T cells where it is in competition with the classical pathway described above mediated by catalytically active PLC-γ1. Interestingly, the BAM32-PLC-γ1-PAK1 pathway to ERK activation is RAS-independent.

We have recently performed an miRNA screen to identify additional signaling pathways in LAT-KI T cells that might contribute to the development and maintenance of lymphoproliferative disease [8]. MicroRNAs (miRNAs) are small (19–22 nucleotide) RNAs that can each regulate expression of a number of other genes [9]. We postulated that some miRNAs might control sets of genes relevant to lymphoproliferative disease in LAT-KI mice. Comparing LAT-KI CD4^+^ T cells to either wild type T cells proliferating in response to *H*. *polygyrus* infection (Th2-dependent) or in response to adoptive transfer to an immunodeficient host (homeostatic proliferation), we identified a small subset of miRNAs that were preferentially expressed in LAT-KI T cells. Of the miRNAs identified, miR-155 has been implicated in a variety of physiological and pathological processes including immune and inflammatory responses, breast cancer, cardiovascular disease, lymphomas and leukemias [10]. In lymphocytes in particular, miR-155 has been shown to regulate function in B cells, CD8^+^ T cells, and in several subsets of CD4^+^ T cells [11].

In this study we explored the possibility that miR-155 could contribute to the pathologic expansion of CD4^+^ T cells in LAT KI mice by influencing the balance between proliferation and apoptosis that contributes to homeostatic equilibrium of T cells. miR-155 can have an indirect effect on BIM-mediated apoptosis. BIM is a pro-apoptotic BH3-only Bcl2 family member that induces apoptosis via the mitochondria. Among Bcl-2 family members, BIM is remarkable in that it can play a major role in mediating apoptosis on its own [12]. For example, Bim/*Bcl2l11* overexpression is sufficient to induce massive T cell death [13]. Bim/*Bcl2l11* expression is regulated by the Forkhead box class O (FOXO) family member FOXO3 [14, 15]. FOXO3 protein levels can be downregulated by miR-155 [16, 17], resulting in a decrease in BIM activity.

AKT and JNK kinases regulate FOXO3 activation in opposite ways [18]. FOXO3 phosphorylation by AKT induces binding of the cytoplasmic chaperones 14-3-3 to FOXO3 and cytosolic sequestration of the FOXO3/14-3-3 complex, preventing FOXO3-mediated Bim/*Bcl2l11* transcription in the nucleus. In contrast, JNK phosphorylation of FOXO3 allows its nuclear translocation, a necessary prerequisite for Bim/*Bcl2l11* transcription. AKT action can be influenced by the phosphoinositide (PI) phosphatase SHIP-1, a primary target of miR-155 [19]. PI dephosphorylation by SHIP-1 prevents AKT and PDK1 activation, which are necessary for activation of the serine/threonine kinase mammalian Target of Rapamycin or mTOR [20, 21].

In this study we show that LAT-KI lymphoproliferative disease is highly dependent on miR-155. BIM-mediated apoptosis was enhanced in LAT-KI T cells upon miR-155 deletion and less lymphoproliferative disease was observed in LAT-KI miR-155 null double mutant mice. We used LAT-KI mice to define T cell signaling pathways, this time downstream from miR-155. Multiple pathways mediated miR-155 regulation of BIM-mediated apoptosis in LAT-KI T cells. FOXO3 activity was influenced directly by miR-155 and indirectly through SHIP-1 and AKT. FOXO3 activity was also controlled by PAK1 and JNK. PAK1 activity was regulated by mTOR, downstream of SHIP-1 and AKT, with mTOR bridging the SHIP-1/AKT and PAK1/JNK pathways. We discuss how miR-155 can affect the balance between the pro-apoptotic PAK/JNK/FOXO3/BIM pathway and the pro-proliferative PAK1/RAF/MEK/ERK pathway to control T cell expansion and death resulting in the regulation of lymphoid organ size and function.

## Materials and Methods

### Mice and ethics statement

LAT Y136F (LAT-KI) mice were backcrossed to C57BL/6 more than ten generations and were described previously [2]. miR155-deficient mice were strain B6.Cg-*Mir155* ^tm1.1Rsky^/J from the Jackson Laboratory. Bim-deficient mice were strain B6.129S1-Bcl2l11^tm1.1Ast^/J from the Jackson Laboratory. Mice were maintained under pathogen-free conditions at an American Association for the Accreditation of Laboratory Animal Care-accredited facility. Mice were housed in accordance with the recommendations in the Guide for the Care and Use of Laboratory Animals of the National Institutes of Health under animal study proposals approved by the NCI-Bethesda Animal Care and Use Committee (ASP#LCMB-013).

### Cell line and cell culture

The human leukemic T cell line Jurkat E6.1 was obtained from American Type Culture Collection (ATCC, Manassas, VA). Jurkat E6.1 cells were cultured in RPMI 1640 supplemented with 10% (vol/vol) heat inactivated fetal bovine serum (FBS), 50 units/ml of penicillin G sodium, 50 **μ**g/ml of streptomycin sulfate (Invitrogen). Cells were maintained at densities between 5x10^5^ and 7x10^5^ cells/ml in a humidified incubator under 5% CO_2_. To activate mTOR kinase, L-leucine, pyruvate, and non-essential amino acids (NEAAs) were added to the culture media. L-leucine (2.5 or 5 mM, L8000) was from Sigma-Aldrich. Sodium pyruvate (1 mM, 11360–070) and NEAAs in MEM medium (1/100, 11140–050) were from Life Technologies.

### Analysis of mouse T cells by flow cytometry

Single cell suspensions of thymus, lymph node (LN), and spleen were stained for cell surface proteins with fluorescently conjugated antibodies (BD Biosciences) and were analyzed on a FACSCalibur (BD Biosciences). Data were analyzed using FlowJo software (TreeStar Inc.). Data are presented gated on live cells as determined by FSC vs. SSC unless otherwise indicated. Annexin-V and 7-AAD staining was performed according to the manufacturer’s instructions (BD Biosciences). Intracellular cytokine and BIM staining were performed on cells that had been fixed and permeabilized using the BD Cytofix/Cytoperm Plus kit. For cytokine staining, cells were pretreated with PMA (Sigma, 50 ng/ml) and ionomycin (Sigma, 750 ng/ml) for a total of 6h and with GolgiStop (BD Biosciences) for the final 4h. Fluorescent anti-cytokine Abs were from BD Biosciences. Anti-BIM Ab was from Cell Signaling and secondary Alexa Fluor 647 goat anti-rabbit IgG was from Life Technologies.

### *In Vivo* T Cell Proliferation

To assess BrdU incorporation of T cells in vivo, mice received 0.8 mg BrdU in PBS by intaperitoneal injection at 0 and 6 h. At 24 h after the initial injection, single cell suspensions were made from LN. LN cells were stained with anti-CD4-APC and anti-BrdU-FITC using the BrdU Flow kit (BD Biosciences). Percentages of CD4^+^ T cells also positive for BrdU were determined by flow cytometry [5].

### Isolation and stimulation of murine CD4^+^ T lymphocytes

Total mouse cells from LN single cell suspensions were isolated using a Dynal mouse CD4 negative isolation kit (Miltenyi Biotec) according to the manufacturer’s instructions. Cells were >85% CD4^+^ following purification. Purified CD4^+^ LN cells were then resuspended in pre-warmed RPMI 1640 at 1x10^6^ cells/10 **μ**l and allowed to equilibrate to room temperature (RT) for 20 min. before any additional manipulation. For stimulation of purified CD4^+^ lymphocytes, purified cells were resuspended in pre-warmed RPMI 1640 at 1x10^6^ cells/10 **μ**l. For each time point, 4x10^6^ cells were pre-incubated with 10 **μ**g/ml biotinylated anti-CD3**ε** (145-2C11, BD Biosciences) with or without 10 **μ**g/ml biotinylated anti-CD4 (GK1.5, BD Biosciences) for 15 min. at RT. Cells were then washed with RPMI 1640 and resuspended at 1x10^6^ cells/10 **μ**l prior to the addition of 40 **μ**l 2X streptavidin (20 **μ**g/ml final concentration). Stimulation was terminated by the addition of 2X SDS sample buffer containing 100 mM DTT and boiling for 6 min.

### Retroviral expression of miR155 in mouse CD4^+^ T cells

The retroviral expression vector pMSCV-miR155 was made by inserting an approximately 200 bp EcoR1-XhoI PCR fragment containing the miR155 stem loop region into the MSCV-IRES-GFP vector (gift of Robert Lewis, University of Nebraska). The PCR fragment was generated with the following primers: Forward, TGCAGGGAATTCAAACCAGGAAGGGGAAGTGT and Reverse, ACATCTCTCGAGATCCAGCAGGGTGACTCTTG. pMSV-miR155 was transfected into Eco Phoenix cells (National Gene Vector Biorepository) using calcium phosphate transfection for production of viral particles as previously described [22]. 45 **μ**m-filtered viral particles were used to infect mouse CD4^+^ T cells that had been previously expanded in culture in media containing IL-2 (50 units/ml), IL-7 (1 ng/ml) and Dynabeads Mouse T-activator CD3/CD28 beads (Life Technologies). Infection was by spinoculation in the presence of 8 **μ**g/ml polybrene (Sigma). After overnight incubation with virus, cells were washed and incubated an additional 3–5 days in media containing IL-2 and IL-7.

### Plasmids

Flag-Pak1 (EX-T0586-M12) and Bam32-YFP (EX-L0180-M16) were purchased from GeneCopoeia. Catalytically dead PLC-**γ**1^CI^-HA (H335F), described previously [7, 23], was a gift from Karen DeBell.

### Protein expression in Jurkat E6.1 T cells

For protein expression Jurkat E6.1 T cells (maximum of 10x10^6^ cells/cuvette) were transfected with 10 μg plasmid cDNA using the electroporation system, solution T (VPA-1002), and program H-10 from Amaxa/Lonza. To minimize cell mortality, antibiotics were added to the culture media only 5 hours post-transfection.

### siRNA reagents and knock-down experiments

All the siRNA buffers used for knocking-down endogenous PAK1, RPTOR, BCL2L11 (Bim), and FOXO3 in Jurkat T cells were from Thermo Scientific/Dharmacon. To knock-down PAK1, ON-TARGET plus SMART pool PAK1 siRNAs (L-003521-00-0005, Thermo Scientific/Dharmacon) were transfected into Jurkat cells using the Amaxa/Lonza system. For RPTOR knock-down, ON-TARGET plus SMART pool Raptor siRNAs (L-004107-00-0005, Thermo Scientific/Dharmacon) was used. For BIM knock-down, SignalSilence BIM siRNA II (6518, Cell Signaling Technology) was used. For FOXO3 knock-down, SignalSilence FOXO3a siRNA II (6303, Cell Signaling Technology) was used. For each depletion, control siRNAs were used in parallel (ON-TARGET plus Non-targeting Pool; D-001810-10-05 from Thermo Scientific/Dharmacon).

### Immunoprecipitation and Immunoblotting

Detailed protocols for immunoprecipitation and immunoblotting are included in S1 Text. In brief, Jurkat E6.1 cells (20-25x10^6^) transiently transfected or not either with the indicated plasmid(s) or siRNAs were then treated or not with the indicated drugs and cultured for the indicated times. At the end of the experiment, cells were washed and pelleted with RPMI + HEPES. Pelleted cells were then lysed in 600 **μ**l of ice-cold lysis buffer (25 mM Tris-HCl pH 8.0, 150 mM NaCl, 5 mM EDTA, 5 mM EGTA, 10 mM sodium pyrophosphate, 5 mM Na_3_VO_4_, 10 mM NaF, 1% Brij 97, 0.5% Octyl-**β**-D-glucoside supplemented with complete protease inhibitor tablets from Roche). Lysates were clarified at 12,000 g for 20 min at 4°C to remove detergent-insoluble material. Soluble material was pre-cleared with 4 **μ**g of normal rabbit IgG bound to 20 **μ**l of Protein A/G Plus-Agarose beads for 1 h at 4°C. The pre-cleared samples were incubated with rotation for 2 h at 4°C with appropriate antibodies previously conjugated to 20 **μ**l Protein A/G Plus-Agarose beads. After washing and elution, samples were analyzed by SDS-PAGE. After transfer to PVDF membranes, membranes were blocked for 1 h at RT with TBST (10 mM Tris (pH 8.0), 150 mM NaCl, and 0.05% Tween 20) with 5% non-fat dry milk and 1% BSA. Membranes were incubated overnight at 4°C with primary Abs diluted in TBST with 5% non-fat dry milk, 1% BSA, followed by a 60 minute incubation at RT with the appropriate secondary Ab-HRP diluted in TBST with 5% milk, 1% BSA (1/20,000). The WBs were then visualized by enhanced chemiluminescence (ECL) using 1 vol of SuperSignal West Pico ECL plus 1/10 vol of SuperSignal West Femto ECL from Pierce.

### FOXO3 subcellular localization

Study of FOXO3 nuclear translocation was performed as described in [24]. Nuclear and cytoplasmic extracts were prepared with a cytoplasmic lysis buffer (10 mM HEPES, 10 mM KCl, 2 mM MgCl_2_, 1 mM DTT) followed by a nuclear lysis buffer (20 mM HEPES, 420 mM NaCl, 1.5 mM MgCl_2_, 250 mM EDTA, 25% glycerol). For all lysis buffers, fresh protease inhibitor tablets (Roche) and 1 mM sodium orthovanadate were added immediately before use. Lamin B and beta-tubulin were used as nuclear and cytoplasmic markers, respectively.

### Preparation of mitochondria-free cytosol for detection of cytochrome C release

Cytochrome C release was performed as described in [25]. Jurkat cells were collected by centrifugation at 300 *g* for 5 minutes at 4°C and washed with ice-cold PBS. The cell pellets were then resuspended in 500 **μ**l of lysis buffer (20 mM HEPES (pH 7.5), 210 mM sucrose, 70 mM mannitol, 1.5 mM MgCl_2_, 10 mM KCl_2_, and 10 **μ**M digitonin supplemented with protease inhibitor tablets). After a 10 minute incubation at 25°C, the samples were spun at 14,000 *g* for 15 minutes, and the mitochondria-free supernatants containing cytosolic proteins were analyzed by SDS-PAGE/WB. Presence of cytochrome C indicated apoptotic cells.

## Results

### miR-155 deficiency curtails LAT-KI lymphoproliferative disease

**Fig 1:**
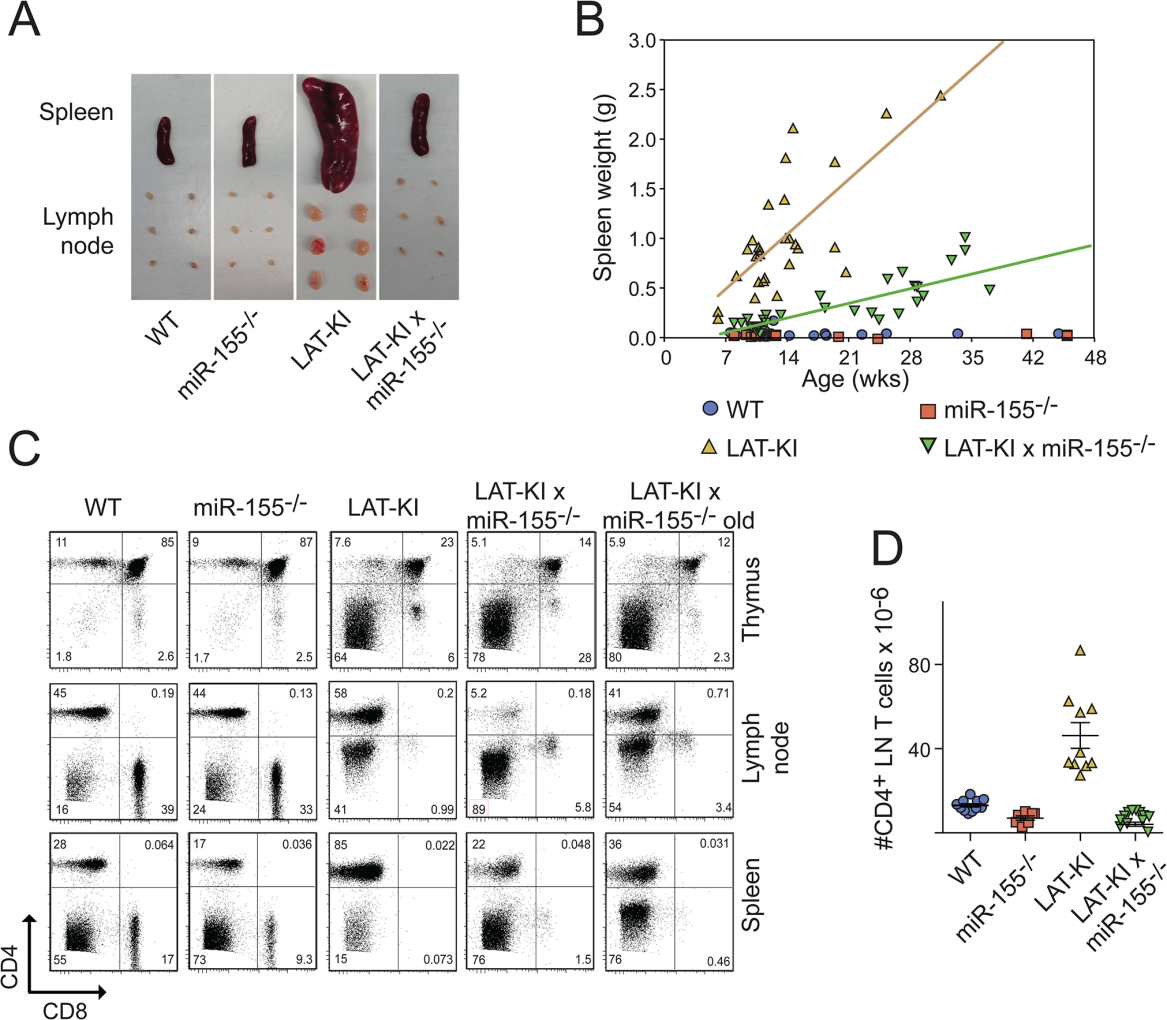
miR-155 deficiency delays lymphoproliferative disease in LAT-KI mice. (A) Photographs of spleens and axillary, brachial and inguinal lymph nodes from WT (C57BL/6), miR155^**-/-**^, LAT-KI and DM (LAT-KI/miR155^**-/-**^) mice. Ages of the mice were 9 wks (WT) and 8 wks (other three genotypes). Photographs are representative of 9 experiments. (B) Spleen weights of mice of the indicated genotypes over time. Each symbol represents an individual mouse. (C) CD4 and CD8 surface marker expression as measured by flow cytometry. Ages of the mice were 11 wks (WT), 9 wks (miR155^**-/-**^), 10 wk (LAT-KI), 10 wks (DM) and 19 wks (DM-old). The results are representative of 9 experiments. (D) Number of CD4^**+**^ T cells from axillary, brachial and inguinal lymph nodes of 8–10 wk old mice of the indicated genotypes. Each symbol represents an individual mouse.

In a previous screen of miRNAs expressed in LAT-KI T cells, we observed overexpression of miR-155 in LAT-KI CD4^+^ T cells compared to WT CD4^+^ T cells proliferating in response to infection by *H*. *polygyrus* or in response to transfer to an immunodeficient host [8]. To determine whether miR-155 influences lymphoproliferative disease in LAT-KI mice or was simply a marker of T cell activation, we interbred miR-155^-/-^ mice with LAT-KI mice to yield double mutant LAT-KI/miR155^-/-^ (DM) mice. One measure of lymphoproliferative disease in LAT-KI mice is spleen weight. As shown for 8-week-old mice (Fig 1A) and over time (Fig 1B), spleen weights in DM mice were much less than in LAT-KI mice. Spleen weights in DM mice increased over time, but at a much slower rate than in LAT-KI mice. LAT-KI mice have a block in thymocyte development at the immature CD4^-^CD8^-^ stage. DM mice showed a similar block in T cell development (Fig 1C). Peripheral T cells in LAT-KI mice are primarily CD4^+^. In DM mice, a smaller percentage of CD4^+^ T cells and a larger percentage of CD8^+^ T cells were present in peripheral lymphoid organs compared to in LAT-KI mice (Fig 1C). In older DM mice, a higher percentage of CD4^+^ T cells was observed, similar to what is seen in younger LAT-KI mice. In addition the absolute numbers of CD4^+^ lymph node T cells were much smaller in DM mice than in LAT-KI mice (Fig 1D). Therefore although lymphoproliferative disease does develop in DM mice (in the absence of miR-155), it does so at a greatly reduced rate.

LAT-KI T cells express large amounts of multiple cytokines including the Th2 signature cytokine, IL-4. Th2 cytokines are produced in response to extracellular parasites, allergens, and toxins whereas Th1 responses are to intracellular bacteria and protozoa. Strong Th2 responses can lead to lung airway inflammation. LAT-KI mice display multiorgan lymphocyte infiltration, especially in the lung [2]. Representative photos from H&E-stained sections of lungs from 11-week-old LAT-KI and DM mice showed substantial perivascular lymphocyte infiltration in the lungs of LAT-KI, but not DM mice (S1 Fig). Consistent with a Th2 phenotype, LAT-KI CD4^+^ lymph node T cells produced IL-4 (S1 Fig)[3]. In addition, some LAT-KI CD4^+^ lymph node T cells produced both IL-4 and IFNγ, the Th1 signature cytokine. Some CD4^+^ lymph node T cells from DM mice also were IL-4 or IL-4/IFNγ-producers (although fewer than in age-matched LAT KI mice), but DM CD4^+^ T cells had more single IFNγ-producing cells. Thus, DM CD4^+^ T cells have more Th1 character than LAT KI CD4^+^ T cells and do not infiltrate the lungs as readily as LAT-KI CD4^+^ T cells, providing more evidence for reduced KI-type lymphoproliferative disease in DM mice.

### Increased basal activation and proliferation of DM CD4^+^ T cells

**Fig 2:**
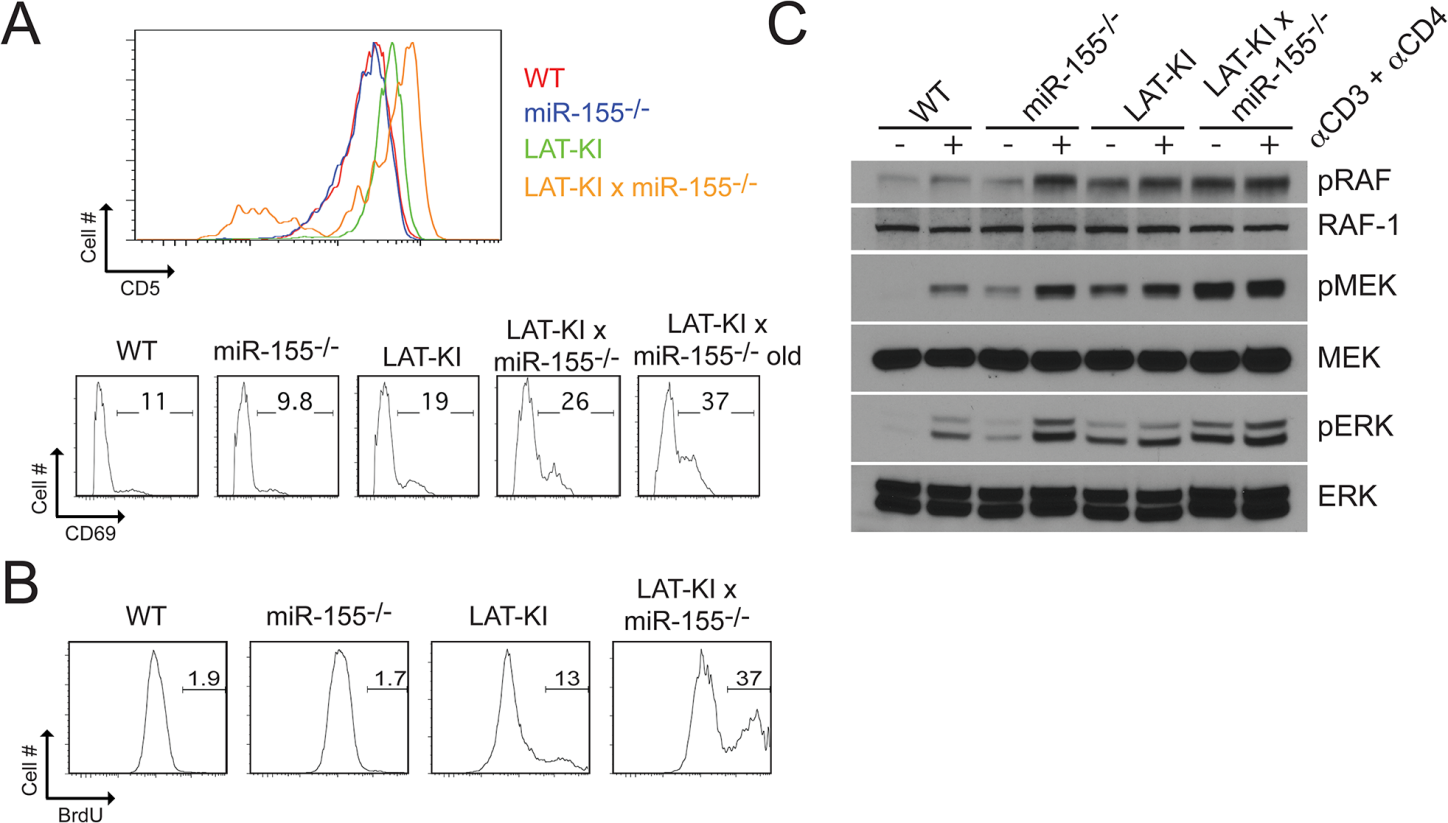
miR-155 deficiency results in increased basal CD4^+^ T cell activation and proliferation in LAT-KI mice. (A) CD5 and CD69 surface marker expression as measured by flow cytometry in CD4^**+**^ lymph node T cells of mice of the indicated genotypes. Ages of the mice were the same as in Fig 1C. The results are representative of 9 experiments. **(**B) In vivo BrdU labeling. CD4^**+**^ lymph node T cells from mice previously injected with BrdU. Ages of the mice were 10 wks (WT) and 8 wks (other 3 genotypes). The results are representative of 3 experiments. **(**C) Negatively selected CD4^**+**^ T cells from the indicated genotypes were stimulated with αCD3ε/CD4 (10 μg/ml, 3 min.). Ages of the mice were 10 wks (WT, miR155^**-/-**^), 11 wks (LAT-KI), and 12 wks (DM). SDS whole cell lysates (WCLs) were analyzed by western blotting (WB). A representative WB is shown (n = 3).

We previously reported that LAT-KI mice show elevated basal ERK activation that is instrumental to their lymphoproliferative phenotype, and that introduction of null mutations of either Bam32 or RasGRP lead to reduced lymphoproliferative disease in LAT-KI mice [5, 6]. These two molecules are components of two separate pathways that lead to ERK activation in LAT-KI T cells. In double mutant BAM32^-/-^LAT-KI [5]or RASGRP1^-/-^LAT-KI T cells [6], proliferation and elevated basal ERK activity were decreased as were levels of markers of T cell activation, CD5 and CD69, when compared to LAT-KI T cells. In contrast, the DM T cells analyzed here showed a completely different phenotype. LAT-KI CD4^+^ T cells expressed elevated levels of CD5 and higher percentages of CD69^hi^ cells than WT CD4^+^ T cells [2, 3]. Surprisingly, although there are fewer CD4^+^ T cells in DM compared to LAT-KI mice, DM CD4^+^ T cells expressed even higher levels of CD5 and higher percentages of CD69^hi^ cells than CD4^+^ T cells from age-matched LAT-KI mice (Fig 2A). We also observed a higher percentage of proliferating CD4^+^ T cells from DM mice compared to age-matched LAT-KI mice as measured by in vivo BrdU incorporation (Fig 2B). Therefore, the reduced number of CD4^+^ T cells in DM mice appeared to be more activated and were proliferating at a higher rate than CD4^+^ T cells from LAT-KI mice. These paradoxical observations led us to attempt to characterize signaling pathways downstream of miR-155 that might affect lymphoproliferative disease.

To analyze activation states of important signaling molecules in freshly isolated, purified CD4^+^ T cells, we utilized western blotting detection of phospho-proteins following TCR stimulation. miR-155 deficiency resulted in increased RAF, MEK and ERK activation in both WT and LAT-KI backgrounds (Fig 2C). Although miR155 KO CD4^+^ T cells showed more MAPK activation upon soluble anti-TCR stimulation than WT CD4^+^ T cells, their ex vivo CD5 and CD69 levels (Fig 2A) and their in vivo proliferative capacities (Fig 2B) were similar to WT. Therefore, only in the stimulatory environment of the LAT KI mouse did miR-155 deficiency correlate with in vivo activation as measured by elevated activation markers and proliferation.

### Increased basal apoptosis in DM CD4^+^ T cells

**Fig 3:**
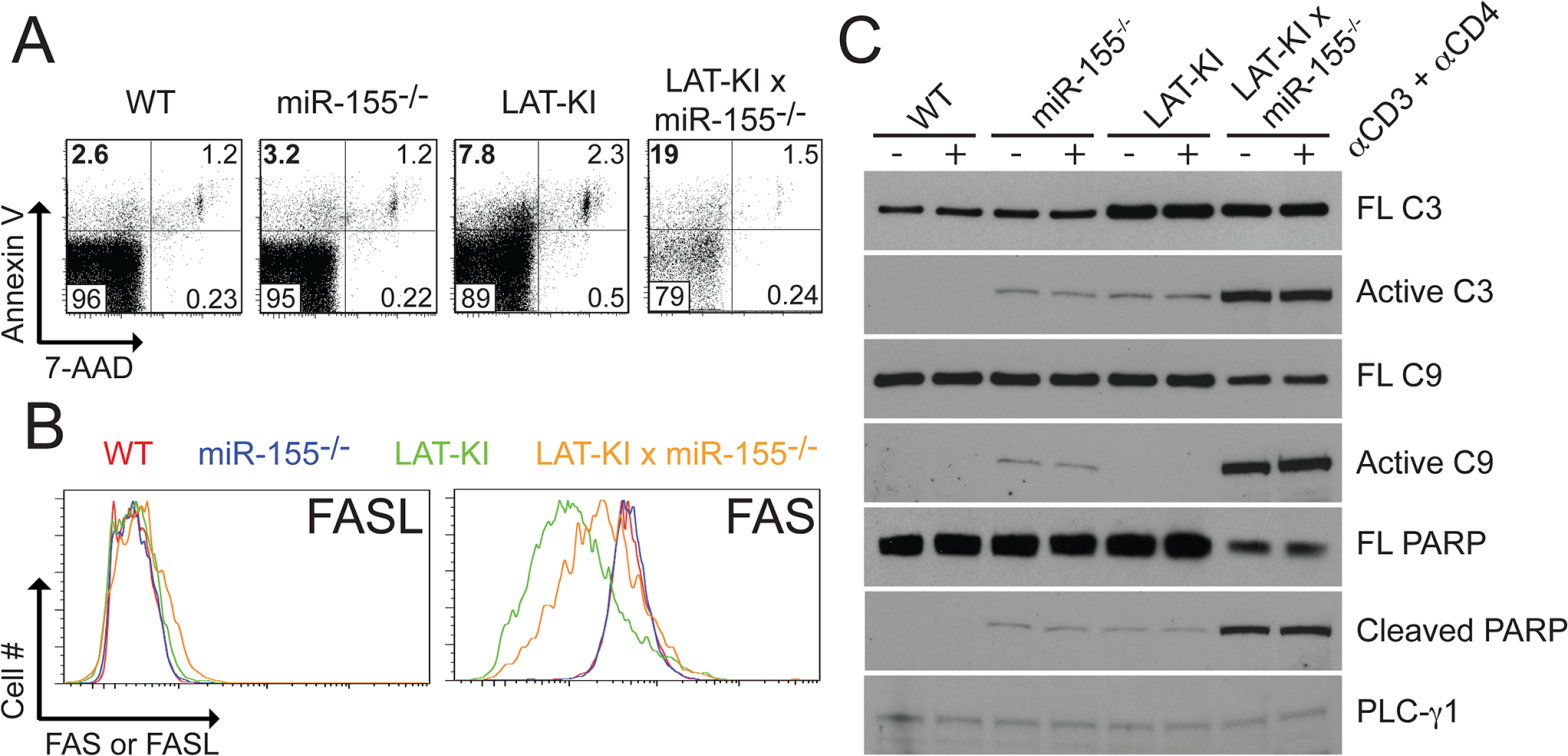
miR-155 deficiency results in increased basal apoptosis in LAT-KI T cells. (A) Apoptotic (Annexin-V^**+**^/7-AAD^**-**^) lymph node CD4^**+**^ T cells as measured by flow cytometry. Ages of the mice were as in Fig 1C. The results are representative of 8 experiments. **(**B) FAS and FAS ligand surface expression as measured by flow cytometry in CD4^**+**^ lymph node T cells of mice of the indicated genotypes. Ages of the mice were as in Fig 1C. The results are representative of 3 experiments. **(**C) Negatively selected CD4^**+**^ T cells from the indicated genotypes were stimulated with αCD3ε/CD4 (10 μg/ml, 3 min). Ages of the mice were 11 wks (WT), 10 wks (miR155^**-/-**^), 11 wks (LAT-KI), and 12 wks (DM). SDS WCLs were analyzed by WB (n = 3). FL indicates full length, C3 Caspase 3 and C9 Caspase 9.

Lymphocyte homeostasis results from a balance between proliferation and apoptosis. Because DM CD4^+^ T cells showed a higher rate of in vivo proliferation than LAT-KI CD4^+^ T cells in spite of having many fewer DM CD4^+^ T cells than LAT-KI CD4^+^ T cells, we also examined whether apoptosis was altered in these cells. Basal apoptosis was measured by ex vivo AnnexinV/7AAD staining. In CD4^+^ T cells from age-matched animals, percentages of AnnexinV^+^/7AAD^-^ cells were higher in DM than in LAT-KI mice, suggesting that miR-155 plays a role in preventing programmed cell death in the LAT-KI background (Fig 3A). We next studied possible mediators of apoptosis in miR-155-deficient T cells. Apoptosis is classically thought to be mediated by both extrinsic and intrinsic pathways [12]. The extrinsic pathway involves activation of FAS by FASL and culminates with Caspase 8 and subsequent Caspase 3 activation. We measured cell surface levels of FAS and FASL by flow cytometry (Fig 3B). FAS levels were lower in LAT-KI and DM CD4^+^ T cells than in WT and miR155^-/-^ CD4^+^ T cells, but FASL levels remained low in all four genotypes. Because FASL levels were low and FAS levels did not correlate with the extent of apoptosis as measured by AnnexinV^+^/7AAD^-^ cells, the increased levels of apoptosis seen ex vivo in DM compared to LAT-KI T cells (and in LAT-KI compared to WT T cells) did not seem to be due to differences in proximal elements of the extrinsic pathway.

The intrinsic pathway of apoptosis is mediated by cell stress that eventually disrupts normal mitochondrial function by increasing levels of mitochondrial pro-apoptotic molecules or by lowering levels of anti-apoptotic molecules [26]. In T cells, the BH3-only protein BIM is a limiting pro-apoptotic molecule, which plays a critical role in the balance of pro-apoptotic versus anti-apoptotic molecules [12]. Indeed, BIM levels predominantly determine cell fate by regulating the balance of survival versus apoptosis in T cells [27–29]. BIM-induced cell death culminates with Caspase 9 and subsequent Caspase 3 activation. We measured Caspase 9 and Caspase 3 activation following anti-CD3/anti-CD4 stimulation of purified CD4^+^ T cells (Fig 3C). Low levels of active Caspases 3 and 9 were detected in miR-155-deficient CD4^+^ T cells. LAT-KI T cells also had low levels of Caspase 3. In the LAT-KI background, miR-155 deficiency resulted in large increases in the levels of active Caspases 3 and 9 and in the levels of cleaved PARP, a Caspase 3 substrate commonly used as a marker of apoptosis. We observed but cannot explain why the LAT-KI mutation and the miR-155 null mutation have synergistic effects on active Caspase 3 and 9 levels. The Caspase and PARP results support a role for the intrinsic pathway in the high basal AnnexinV staining seen in DM T cells. The results also suggest that an upstream mitochondrial pro-apoptotic factor may be up-regulated upon miR-155 deletion and that the action of this factor may lead to enhanced apoptosis in the LAT-KI background.

### A miR-155/FOXO3/BIM proapoptotic pathway

**Fig 4:**
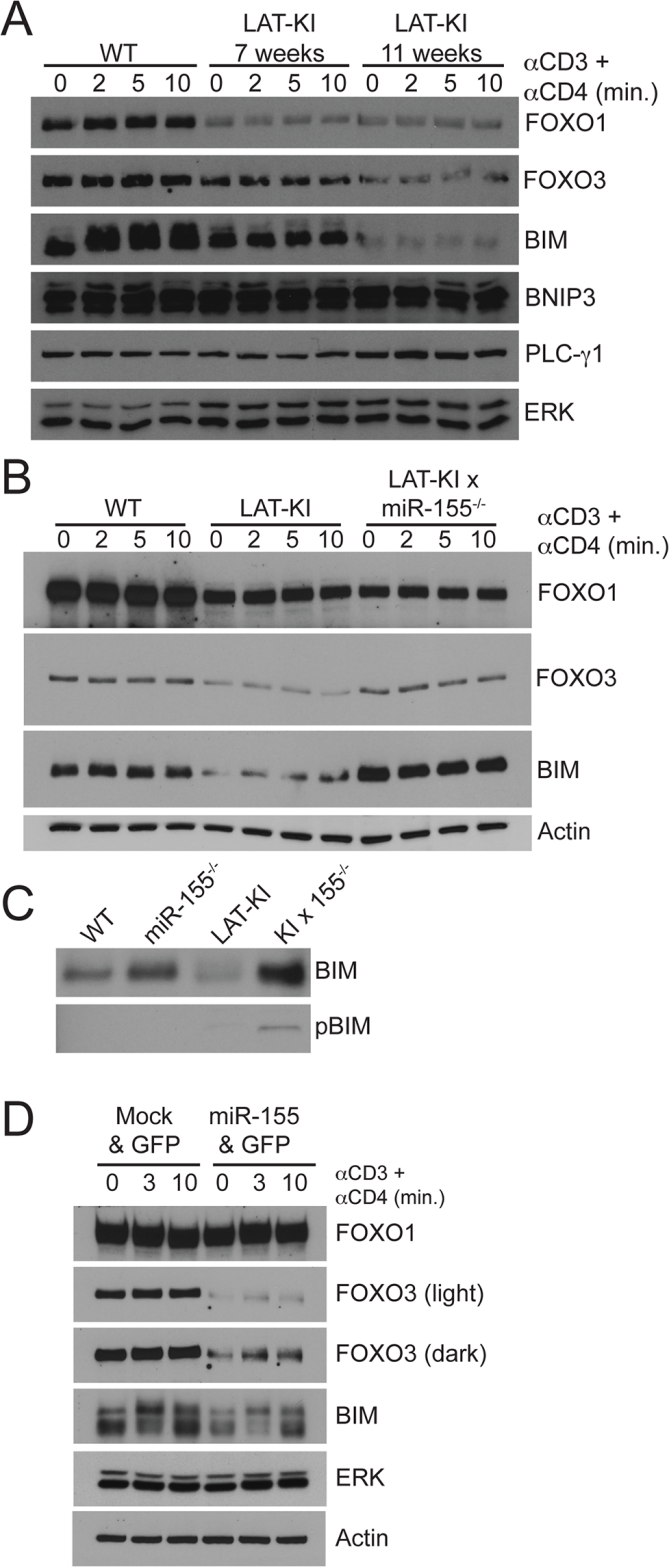
miR-155 negatively regulates the transcription factor FOXO3, which positively regulates the pro-apoptotic factor BIM. (A) Negatively selected CD4^**+**^ T cells from WT and LAT-KI mice were stimulated with αCD3ε/CD4 (10 μg/ml) for the indicated times. Ages of the mice were 11 wks (WT), 7 and 11 wks (LAT-KI young and old). SDS WCLs were analyzed by WB (n = 5). (B) CD4^**+**^ T cell purification, stimulation and WB were as in Fig 4A. Ages of the mice were 11 wks (WT, LAT-KI) and 14 wks (miR155^**-/-**^). SDS WCLs were analyzed by WB (n = 3). (C) CD4^**+**^ T cell purification, stimulation and WB were as in Fig 4A. Ages of the mice were 9 wks (WT), 12 wks (miR155^**-/-**^, LAT-KI), and 14 wks (DM). SDS WCLs were analyzed by WB (n = 3). (D) miR-155 was overexpressed in mouse CD4^**+**^ cells by retroviral infection. Mock infection was performed as a negative control. In both cases, GFP was expressed to identify infected cells. Sorted GFP^**+**^CD4^**+**^ T cells were stimulated with αCD3ε/CD4 (10 μg/ml) for 0, 3 or 10 min. SDS WCLs were analyzed by WB (n = 2).

Among known targets of miR-155 potentially involved in apoptosis, the forkhead transcription factor FOXO3 was of interest because it induces transcription of the pro-apoptotic factor Bim/*Bcl2l11* [14, 30–32]. Because BIM is essential for control of immune system homeostasis and BIM levels critically determine CD4^+^ T cell fate by regulating the balance between cell survival and cell death, we hypothesized that loss of miR-155 regulation of a FOXO3/BIM pathway might be responsible for increased apoptosis in DM compared to LAT-KI T cells and decreased lymphoproliferative disease in DM compared to LAT-KI mice. The fatal lymphoproliferative disease affecting LAT-KI mice increases with age, therefore we investigated FOXO3 and BIM levels in young (7 weeks old) and older (11 weeks old) LAT-KI mice (Fig 4A). FOXO3 levels were diminished in young LAT-KI mice compared to WT and the decrease was even more pronounced as the lymphoproliferative disease advanced. In line with decreased FOXO3 levels, BIM levels were low in LAT-KI T cells and decreased more with age, supporting a role for FOXO3 in Bim/*Bcl2l11* synthesis in LAT-KI CD4^+^ T cells. Expression of BNIP3, another BH3-only pro-apoptotic molecule [33], was not affected by age in LAT-KI T cells.

To investigate a role for miR-155 in modulating levels of FOXO3 and subsequently BIM, we compared FOXO3 and BIM levels in CD4^+^ T cells from LAT-KI and DM mice (Fig 4B). Deletion of miR-155 in the LAT KI background restored FOXO3 to wild-type levels and BIM to greater than wild-type levels. Levels of FOXO1, a forkhead transcription factor that is not known to be regulated by miR-155 [34], were unchanged by miR-155 deletion in the LAT-KI background. In both WT and LAT-KI backgrounds, BIM levels were increased as a result of miR-155 deficiency (Fig 4C). Phospho-BIM (indicative of increased apoptotic tendency,[35]) was only detectable in DM T cells (Fig 4C). In addition, overexpression of miR-155 in WT CD4^+^ T cells decreased both FOXO3 and BIM levels (Fig 4D) confirming a downstream effect of miR-155 on FOXO3 and BIM.

### Regulation of the miR-155/FOXO3/BIM pathway by SHIP-1/AKT

A second well known target of miR-155 is the phosphatidylinositide phosphatase SHIP-1 [19]. SHIP-1 activity opposes the action of PI3 kinase (PI3K), which catalyzes the conversion of PIP2 to PIP3 following T cell activation. SHIP-1 catalyzes the conversion of PIP3 to PI(3,4)P2. Because PIP3 recruits the serine threonine kinase AKT to the plasma membrane via its PH domain, SHIP-1 downregulates AKT activity [36]. SHIP-1 downregulation of AKT activity has been demonstrated in Jurkat T cells [37]. 3-phosphoinositide-dependent kinase 1 (PDK1) is also recruited to the plasma membrane via its PH domain and phosphorylation of AKT by PDK1 allows for the activation of AKT. Some downstream targets of AKT are mTOR (leading to enhanced cell growth and G1 cell cycle progression via the phosphorylation of p70S6K), GSK3, the pro-apoptotic factor BAD, and transcription factors FOXO1 and FOXO3 [38]. AKT phosphorylation of FOXO3 allows its association with 14-3-3 dimers, which leads to cytoplasmic retention of FOXO3, preventing it from acting as a transcription factor [39]. Therefore, deletion of miR-155 would be predicted to sequentially increase SHIP-1 levels, decrease AKT and PDK1 activity, and increase nuclear FOXO3 activity, leading to more BIM-mediated apoptosis.

**Fig 5:**
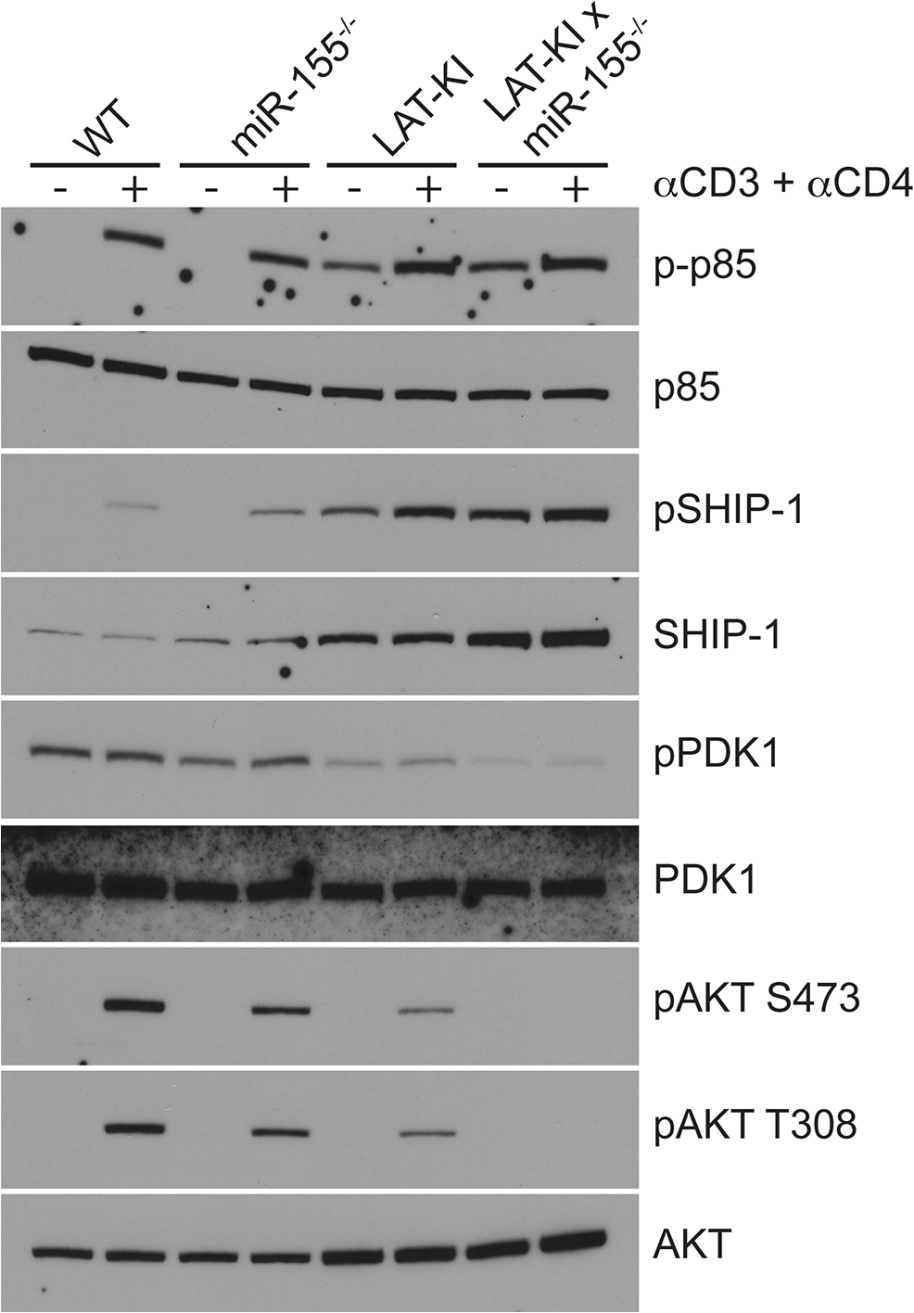
miR-155 deficiency decreases signaling in the PI3K/mTOR pathway. Negatively selected CD4^**+**^ T cells from the indicated genotypes were stimulated with αCD3ε/CD4 (10 μg/ml, 3 min). Ages of the mice were 11 wks (WT), 10 wks (miR155^**-/-**^), 11 wks (LAT-KI), and 12 wks (DM). SDS WCLs were analyzed by WB (n = 3).

We investigated SHIP-1, AKT, and PDK1 expression and phosphorylation in CD4^+^ T cells from miR155-deficient mice in WT and LAT-KI backgrounds (Fig 5). As expected, miR-155 deficiency resulted in increased SHIP-1 levels in both backgrounds. A consequent decrease in AKT activity as measured by AKT phosphorylation at both S473 (site of TORC2 phosphorylation) and T308 (site of PDK1 phosphorylation) [40] was observed as was phosphorylation of PDK1 (Fig 5). Basal PI3K (p85) phosphorylation was higher in LAT-KI-derived T cells than in WT-derived T cells although AKT phosphorylation was lower (Fig 5), suggesting that SHIP-1 (and not PI3K) plays a dominant role in determining AKT phosphorylation levels in LAT-KI T cells. Thus in addition to the direct effect of the loss of miR-155 on increasing FOXO3 levels, the loss of SHIP-1 repression of AKT can also have a parallel or added effect on increasing FOXO3 levels.

### Regulation of the miR-155/FOXO3/BIM pathway by PAK1/JNK

**Fig 6:**
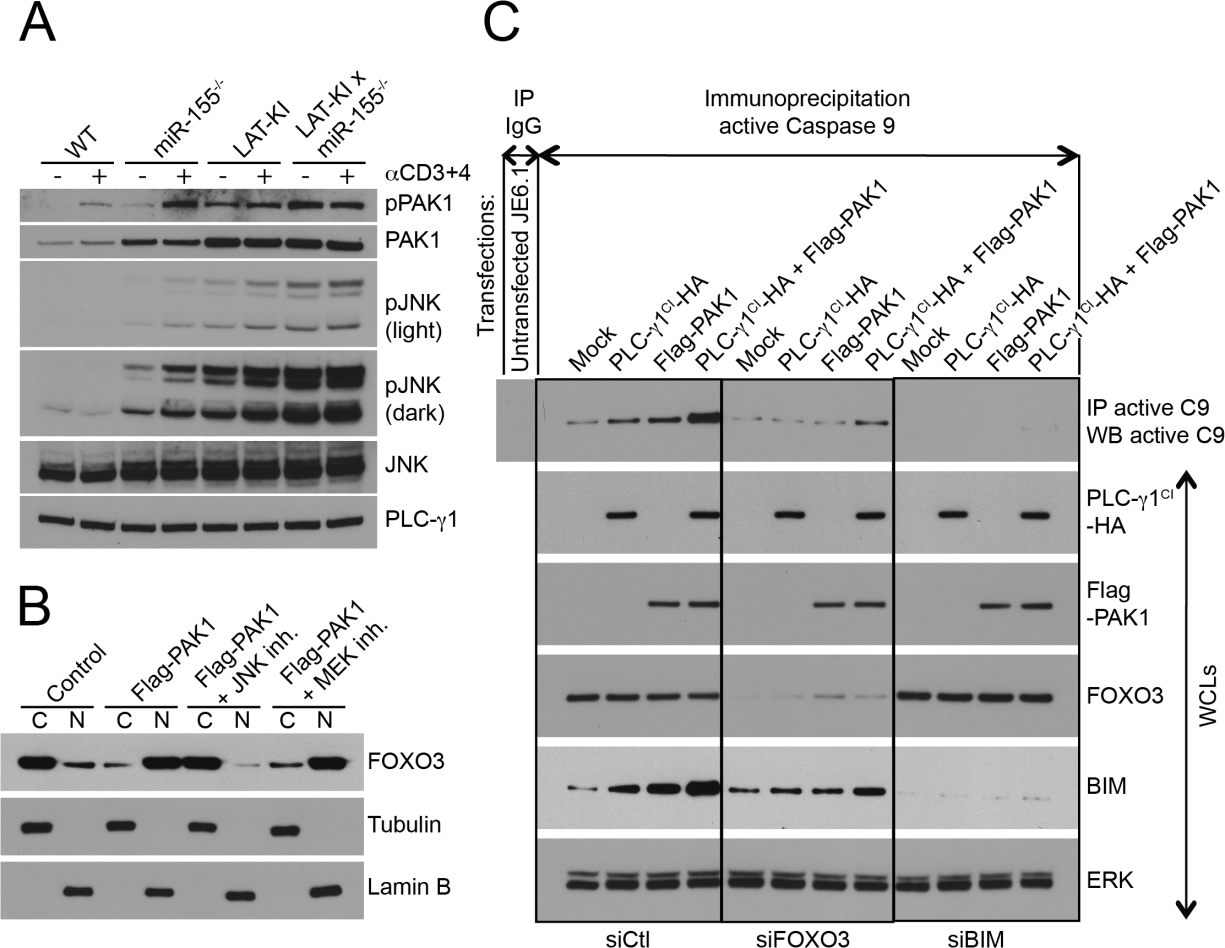
miR-155 deficiency activates PAK1 and a PAK1-nucleated apoptotic pathway (JNK/FOXO3/BIM/Caspase 9). (A) Negatively selected CD4^**+**^ T cells from the indicated genotypes were stimulated with αCD3ε/CD4 (10 μg/ml, 3 min.). Ages of the mice were 11 wks (WT), 10 wks (miR155^**-/-**^), 11 wks (LAT-KI), and 12 wks (DM). SDS WCLs were analyzed by WB (n = 3). (B) Jurkat T cells were transfected with Flag-PAK1 and PLC-γ1^**CI**^-HA cDNAs. 24h post-transfection, cells were treated for 4h with MEK inhibitor (U0126, 20 μM) or JNK inhibitor (SP600125, 20 μM). Lamin B-enriched nuclear and tubulin-enriched cytosolic fractions were prepared. PAK1/JNK-mediated FOXO3 nuclear translocation was studied by WB (n = 3). **(**C) Jurkat T cells were transfected with FOXO3a, BIM/Bcl2l11 or control siRNAs (150 nM). After 24h, cells were super-transfected with siRNAs (150 nM) plus PLC-γ1^**CI**^-HA, Flag-PAK1, or both cDNAs (10 μg). After an additional 36h, cells were lysed. Lysates (75%) were subjected to an active Caspase 9 IP and the 25% remaining lysates were used to make WCLs. Samples were analyzed by WB (n = 3).

Because multiple MAPK pathways affect the growth of LAT-KI T cells, we also wanted to explore whether BIM-mediated apoptosis downstream of FOXO3 could be mediated by MAPKs. The MAP kinase JNK phosphorylates FOXO3 inducing its translocation to the nucleus where it stimulates Bim/*Bcl2l11* transcription [18]. In addition, JNK phosphorylates BIM increasing its capacity to induce caspase-dependent apoptosis [35, 41, 42]. BIM phosphorylation at the JNK site [35] is only apparent in DM T cells (Fig 4C). Accordingly we asked whether JNK activation was affected by miR-155 deficiency in WT and LAT-KI backgrounds. JNK protein levels were not substantially affected by miR-155 deletion. However, phospho-JNK levels were increased by miR-155 deletion in both backgrounds (Fig 6A).

Our previous experiments detailing a role for the serine/threonine kinase PAK1 in MAP kinase activation gave us the means for testing a potential role for PAK1 in JNK activation. In the former studies we demonstrated how PAK1 activation via BAM32-PLC-γ1-PAK1 complexes led to MAPK activation [7]. Here we describe two sets of experiments that indicate that PAK1 activation can lead to JNK activation in T cells. In the first experiments, we overexpressed Bam32/*Dapp1* in Jurkat T cells to achieve PAK1 activation (S2 Fig). Bam32 overexpression led to JNK phosphorylation (in addition to MEK phosphorylation). Knockdown of PAK1 using *Pak1* siRNA prior to Bam32 transfection showed that PAK1 was necessary for the BAM32-induced JNK (and MEK) phosphorylation. In the second set of experiments, in mouse T cells, BAM32 deficiency was used to modulate PAK1 activity (S2 Fig). In both WT and LAT-KI backgrounds, BAM32 deficiency resulted in less PAK1 phosphorylation at T423 (catalytic site). This was expected because BAM32-PLC-γ1-PAK1 complexes cannot form and activate PAK1. Although JNK phosphorylation was below the limit of detection in WT and BAM32 KO T cells (and therefore is not shown), JNK phosphorylation was greatly diminished in LAT-KI BAM32 KO double mutant T cells compared to LAT-KI T cells.

Having determined that PAK1 activation can lead to JNK activation in T cells, we next investigated the effect of miR-155 deficiency on PAK1 levels and phosphorylation (Fig 6A). miR-155 deletion in the WT background (miR155 KO vs WT) resulted in an elevation of PAK1 levels. In LAT-KI T cells, PAK1 protein levels were high and did not increase with miR-155 deletion. Phospho-PAK1 levels were enhanced by miR-155 deficiency in both WT and LAT-KI backgrounds. To further investigate a potential role for miR-155 in controlling PAK1 levels and phosphorylation, we overexpressed miR-155 in WT CD4^+^ T cells using retroviral infection (S2 Fig). Overexpression of miR-155 in these cells resulted in a decrease in PAK1 levels and phosphorylation. In addition phosphorylation of MEK1 at the site of phosphorylation by PAK1 (S298) [43, 44] was decreased with miR-155 overexpression whereas RAF-1 and ERK phosphorylation were not much affected by miR-155 overexpression. Therefore miR-155 deficiency resulted in increased PAK1 levels and phosphorylation whereas miR-155 overexpression resulted in decreased PAK1 levels and activity.

We hypothesize that miR-155 (most likely indirectly) regulates PAK1. In the absence of miR-155, elevated PAK1 activates JNK leading to phosphorylation of FOXO3 and its nuclear translocation and activation, and thus enhanced BIM-mediated apoptosis. Having shown that PAK1 can activate JNK (above), we next showed that PAK1 through JNK, and not through ERK, led to nuclear translocation of FOXO3. We measured the amount of FOXO3 protein in cytosolic and nuclear cellular fractions after PAK1 transfection into Jurkat T cells in the absence or presence of JNK or MEK inhibitors. PAK1 overexpression induced nuclear translocation of FOXO3 and this translocation depended on JNK, and not ERK, activity (Fig 6B, S2 Fig). To further show that PAK1 overexpression led to increased levels of BIM and markers of BIM-mediated apoptosis, in Fig 6C we show that BIM levels increased following transfection of PAK1 into Jurkat T cells, especially in combination with expression of catalytically inactive PLC-γ1 (PLC-γ1^CI^), which favors formation of BAM32-PAK1-PLC-γ1 complexes. Levels of downstream indicators of apoptosis were also enhanced under these conditions including active Caspase 9 (Fig 6C) and cytochrome C (S3 Fig).

To confirm that FOXO3 activity leads to increased levels of BIM and that BIM leads to apoptosis in T cells (as measured by Caspase 9 activity), we performed the knockdown experiments shown in Fig 6C. As described above, we transfected Jurkat T cells with PAK1 in combination with PLC-γ1^CI^ to promote activation of PAK1. In the same experiment we used siRNAs to knockdown FOXO3 or BIM/Bcl2l11 expression. Knockdown of FOXO3 dampened the increases in BIM levels and Caspase 9 activity, suggesting that FOXO3 is indeed upstream of BIM and that there are other contributors to BIM activity. Knockdown of BIM prevented Caspase 9 activation, as expected. Furthermore, Caspase 9 inhibition (shown in S3 Fig) had no effect on BIM levels.

**Fig 7:**
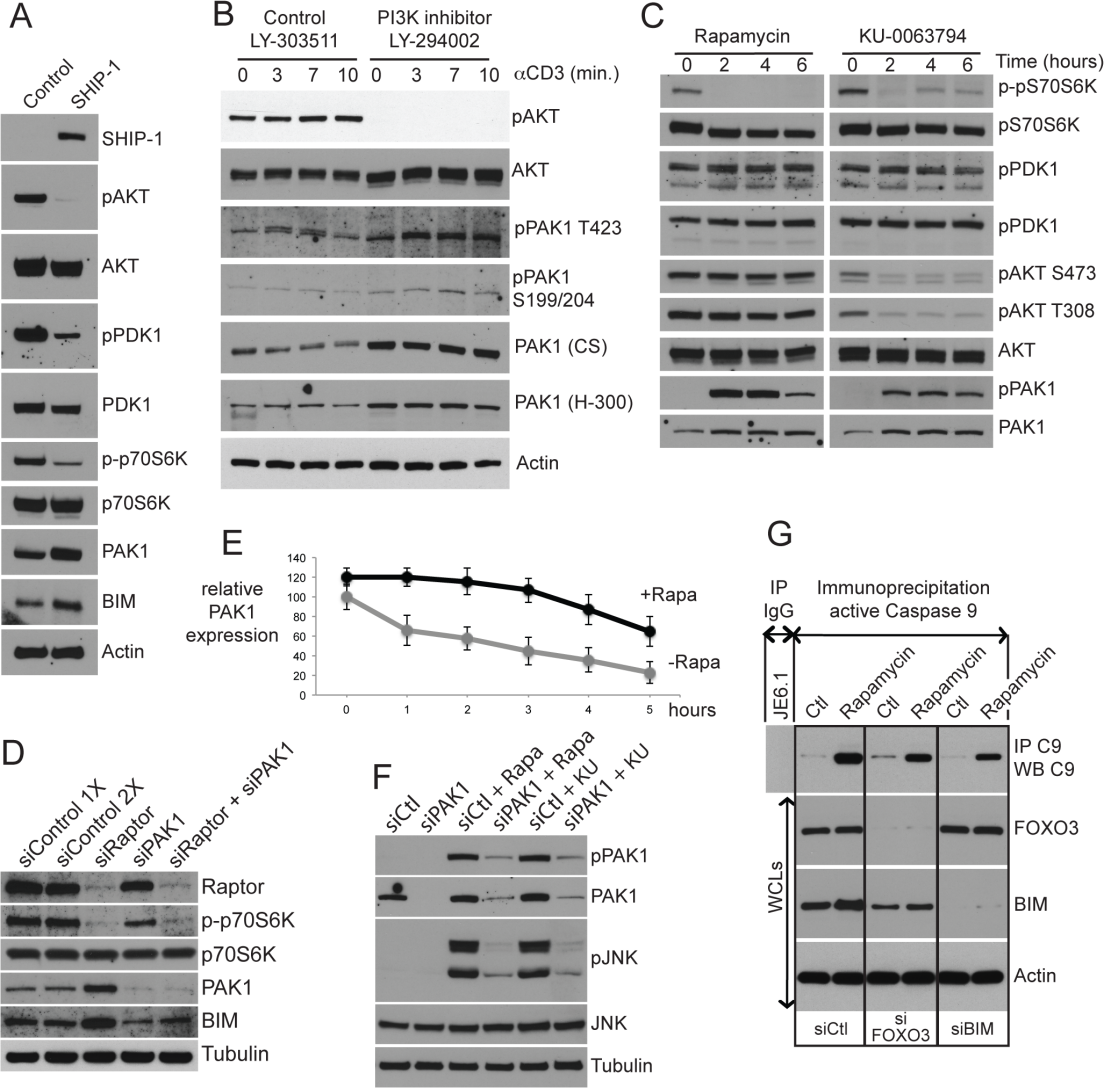
mTOR inhibition activates PAK1 signaling and a PAK1-nucleated apoptotic pathway (JNK/FOXO3/BIM/Caspase 9). (A) Naturally SHIP-1-deficient Jurkat T cells were transfected with SHIP-1 cDNA (10 μg). 24h post-transfection, SDS WCLs were analyzed by WB(n = 4). **(**B) Jurkat T cells pre-treated with PI3K inhibitor (LY-294002, 50 μM) or control inactive enantiomer (LY-303511, 50 μM) for 90 min were stimulated with αCD3 (2 μg/ml) for the indicated times. SDS WCLs were analyzed by WB (n = 3). **(**C) Jurkat T cells were treated with Rapamycin or KU-0063794 (100 nM) for 0, 2, 4, or 6h. SDS WCLs were analyzed by WB (n = 3). **(**D) Jurkat T cells were transfected with Raptor/RPTOR, PAK1, both, or control siRNAs (200 μM). 48h post-transfection, SDS WCLs were analyzed by WB (n = 3). **(**E) Jurkat T cells were first starved (0.5% FCS) for 16h then pre-treated for 2h with cycloheximide (CHX, 50 μg/ml). After a 2h CHX pre-treatment, cells were not washed and Rapamycin (100 nM) was added to the media. Every hour SDS WCLs were made. Samples were analyzed by WB. Quantitation of a representative WB is shown (n = 3). The WB can be found in (B) of S4 Fig. **(**F) Jurkat T cells were transfected with Pak1 or control siRNAs (200 μM). 48h post-transfection, cells were treated with Rapamycin or KU-0063794 (100 nM, 2h). SDS WCLs were analyzed by WB (n = 4). **(**G) Jurkat T cells were transfected with FOXO3, BIM or control siRNAs (200 nM). After 36h, cells were treated with Rapamycin (100 nM, 16h) and lysed. Lysates (75%) were subjected to an active Caspase 9 IP and the 25% remaining lysates were used to make WCLs. Samples were analyzed by WB (n = 3).

We propose that two parallel pathways promote nuclear translocation of FOXO3 and subsequent BIM-mediated apoptosis. One pathway utilizes PAK1/JNK and the other SHIP-1/AKT. SHIP-1 is a direct target of miR-155. PAK1 is not predicted to be a direct target of miR-155 [34]. We therefore wanted to investigate how miR-155 could influence PAK1 activity. We first asked if the effect of miR-155 on PAK1 was phenocopied by SHIP-1 overexpression to see if the effect was through SHIP-1. We tested the effect of SHIP-1 overexpression on PAK1 levels in Jurkat T cells, which are deficient for SHIP-1 [37]. Transfection of Jurkat T cells with SHIP-1 resulted in increased levels of PAK1 (Fig 7A), similar to the effect of miR-155 deletion in WT mouse T cells. As mentioned before, high SHIP-1 levels in DM T cells could result in low pAKT and pPDK1 levels in these cells. SHIP-1 transfection of Jurkat T cells resulted in lower pAKT and pPDK1 levels and higher BIM levels. We next determined if decreasing levels of pAKT would also lead to an increase in PAK1 levels to see if the miR-155 effect on PAK1 was through SHIP-1 and AKT. To accomplish this, we treated Jurkat T cells with a PI3K inhibitor, which caused a dramatic decrease in pAKT levels (Fig 7B). Under these circumstances, PAK1 and pPAK1 levels were increased. These data suggest that low AKT activity correlates with increased PAK1 levels and that the effect of miR-155 on PAK1 is through SHIP-1 and AKT.

### mTOR crosstalk between the SHIP-1/AKT/FOXO3 and PAK1/JNK/FOXO3 pathways

A major target of AKT is mTOR and mTOR is a master regulator of apoptosis [45], which we propose is a major determinant for altering lymphoproliferative disease in DM mice. We hypothesized that mTOR is the connection between the SHIP1/AKT and JNK/PAK1 pathways with AKT acting on mTOR and mTOR regulating PAK1. In miR-155-deficient DM CD4^+^ T cells, SHIP-1 levels are elevated, AKT levels are depressed, and mTOR levels would be predicted to be down. Therefore, to mimic the effect of miR-155 deficiency on mTOR activity, we employed inhibitor and siRNA approaches to downregulate mTOR activity in Jurkat T cells. The high molecular weight of mTOR (289 kDa) renders its detection by western blotting difficult. For this reason, phosphorylation of p70S6K (an mTOR substrate) is often used as a surrogate for the analysis of mTOR activity. mTOR kinase can be part of two different complexes, mTORC1 and mTORC2. The differential roles of mTORC1 and mTORC2 can be studied using the mTOR inhibitor Rapamycin. mTORC1 is specifically inhibited by low concentrations of Rapamycin (100 nM) if the treatment does not extend beyond a few hours. In contrast, KU-0063794 (hereafter referred to as KU) inhibits both mTORC1 and mTORC2. Rapamycin analogs such as Deforolimus, Everolimus, and Temsirolimus have the same binding sites to mTOR as Rapamycin but have more beneficial pharmacokinetics.

To test the hypothesis that mTOR is the connection between AKT and PAK1, we first determined the effect of altering SHIP1 and AKT levels on mTOR activity. Increasing SHIP1 levels and decreasing pAKT levels in Jurkat T cells results in less p-p70S6K, reflecting less mTOR activity (Fig 7A). Next we asked what the effect of decreasing mTOR activity is on PAK1 levels and phosphorylation using two strategies. The first strategy (Fig 7C) used Rapamycin treatment of Jurkat T cells. Under the conditions used here (100 nM for 6 hr), Rapamycin specifically inhibited mTORC1 activity, as confirmed by the absence of detectable p-p70S6K, but not mTORC2 activity, as AKT phosphorylation on both T308 and S473 was unchanged (mTOR in the mTORC2 complex phosphorylates AKT whereas AKT phosphorylates mTOR in the mTORC1 complex). Rapamycin treatment resulted in increased PAK1 and pPAK1 levels. Because AKT and PDK1 are upstream of mTOR, their levels were not affected by Rapamycin treatment. p-p70S6K levels, downstream of mTOR, were decreased, verifying effective Rapamycin treatment. In contrast to Rapamycin treatment, KU treatment (also 100 nM for 6 hr) inhibited both mTORC1 and mTORC2, as confirmed by the absence of AKT phosphorylation on both T308 and S473 as well as p70S6K phosphorylation. The data collectively suggest that decreases in mTORC1 activity preferentially enhanced PAK1 signaling. In S4 Fig, treatment with the rapalogs Deforolimus, Everolimus, and Temsirolimus confirmed the Rapamycin effect. The second strategy that we used to downregulate mTOR activity was treatment with siRNA to Raptor/RPTOR, part of the mTORC1 complex, which also resulted in increased PAK1 levels and downstream BIM levels (Fig 7D). In addition, downstream of PAK1, MEK, ERK and JNK phosphorylation were all enhanced by Rapamycin and Rapalog treatments (Fig 7C and S4 Fig). Therefore, inhibition of mTOR activity resulted in increased PAK1 levels and downstream pERK, pJNK, and BIM levels.

We questioned whether this mTOR effect could be mediated by changes in PAK1 stability. We investigated PAK1 protein stability by treating Jurkat T cells with the protein synthesis inhibitor cycloheximide in the absence or presence of Rapamycin (S4 Fig and Fig 7E). Decreasing mTOR activity using Rapamycin treatment resulted in delayed PAK1 degradation and prolonged PAK1 half-life. We also tested whether increasing mTOR activity could affect PAK1 levels (S4 Fig). To this end, we treated Jurkat T cells with the amino acid leucine, an mTOR activator [46, 47](2.5 or 5 mM for 16 hrs). In addition, we treated cells with commercial cell culture reagents containing non-essential amino acids and pyruvate, alone or in combination. Leucine reduced Pak1 protein levels in a dose-dependent manner. Non-essential amino acids and pyruvate also reduced Pak1 levels, especially in combination. BIM levels followed the same pattern as PAK1 reduction, supporting the existence of a PAK/JNK/FOXO3/BIM pathway. Parenthetically, non-essential amino acids and pyruvate are commonly used to improve cell culture. Their reduction of BIM levels may represent a mechanism for increasing cell viability.

We performed several experiments to verify the cascade, ↓mTOR→↑PAK1→↑JNK→↑FOXO3→↑BIM→↑Caspase9. To investigate the linearity of the mTOR→PAK1→JNK arm, we treated Jurkat T cells with Rapamycin and KU and siRNA to PAK1 (Fig 7F). Inhibition of mTOR (Rapamycin) yielded higher levels of PAK1, p-PAK1 and pJNK. Inhibition of PAK1 by siPAK1 blocked the increase in pJNK seen following treatment with Rapamycin. To investigate the FOXO3→BIM→Caspase 9 arm, Rapamycin treatment was used in conjunction with siRNA treatment to FOXO3 and BIM (Fig 7G). Consistent with the effect of Rapamycin on PAK1 and JNK, downstream BIM and active Caspase 9 levels were increased in response to Rapamycin treatment. siRNA to FOXO3 partially decreased the Rapamycin-induced upregulation of BIM and active Caspase 9 levels. siRNA to BIM partially decreased the Rapamycin-induced upregulation of active Caspase 9.

Finally, to show that PAK1 effects on BIM and active Caspase 9 were mediated through PAK1/JNK and not PAK1/ERK pathways, we used MEK and JNK inhibitors in conjunction with Rapamycin (S4 Fig). Rapamycin treatment led to an increase in BIM and active Caspase 9 levels. Addition of a JNK inhibitor reduced the induction of BIM and active Caspase 9. However, addition of a MEK inhibitor to Rapamycin treatment yielded the same levels of BIM and active Caspase 9 induction as induction by Rapamycin alone. Therefore, we can conclude that the PAK1/JNK pathway, and not the PAK1/ERK pathway, is involved with induction of BIM-mediated apoptosis downstream from mTOR signaling in T cells. Furthermore, we show that PAK1 is required for the Rapamycin-induced effects on BIM and active Caspase 9 using PAK1 siRNAs (compare siControl + Rapamycin to siPAK1 + Rapamycin in S4 Fig). Collectively, these data support a model whereby JNK is activated via PAK1 and PAK1 activity is regulated by mTOR.

**Fig 8:**
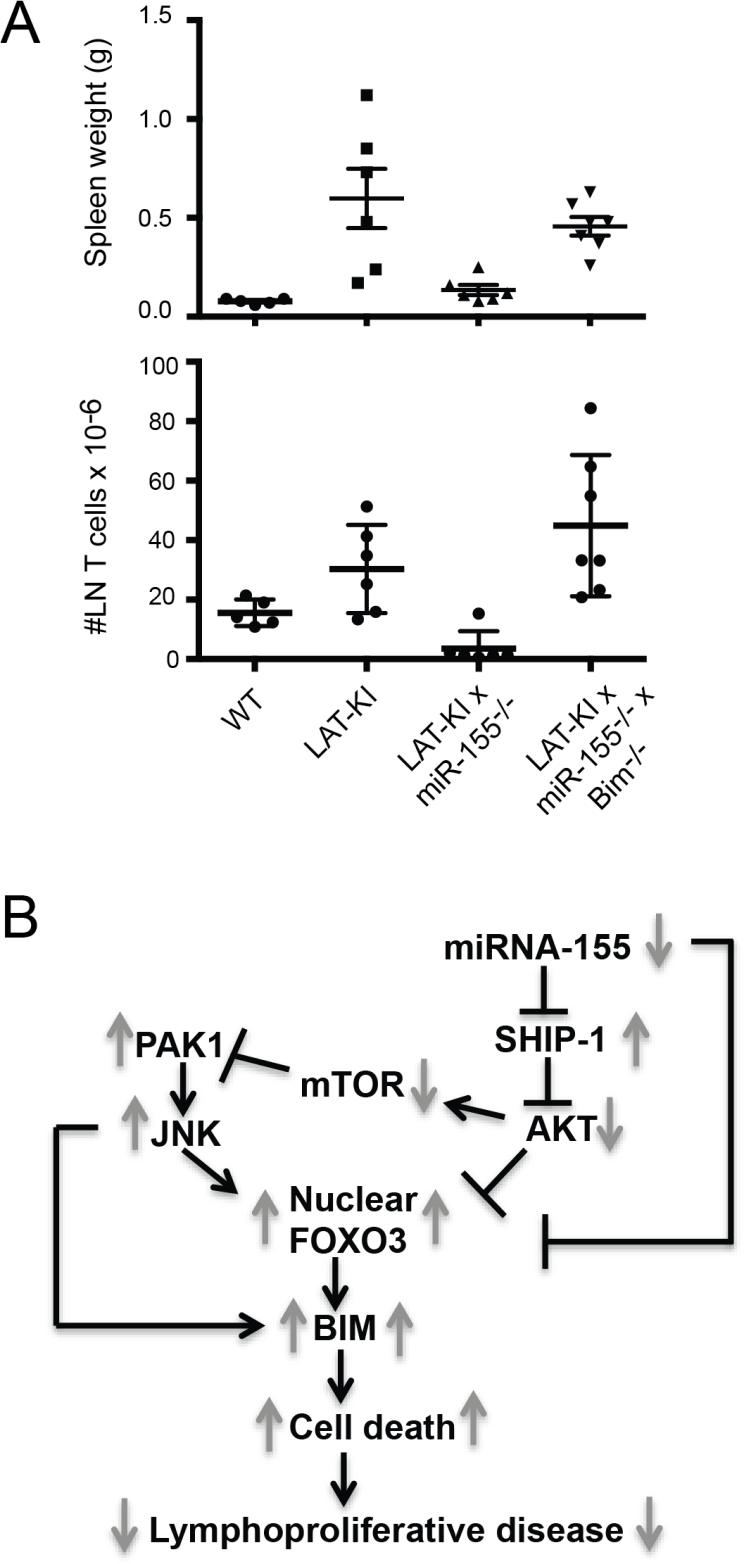
Proposed pathways mediating miR-155 control of lymphoproliferative disease in LAT-KI CD4^+^ T cells act through BIM. (A) BIM deficiency accelerates lymphoproliferative disease in DM mice. Spleen weights of 6–10 wk old mice of the indicated genotypes (upper chart). Number of CD4^**+**^ + CD8^**+**^ T cells from axillary, brachial and inguinal lymph nodes of 6–10 wk old mice of the indicated genotypes (lower chart). Each symbol represents an individual mouse. (B) Proposed pathways mediating miR-155 control of lymphoproliferative disease in LAT-KI CD4^**+**^ T cells. Gray arrows indicate relative action caused by miR-155 deficiency. The function of AKT may be replaced by PDK1.

To summarize our results, miR-155 acts through its direct targets SHIP-1 and FOXO3. FOXO3 nuclear translocation and activation occurs via two pathways, SHIP-1/AKT and PAK1/JNK. mTOR provides crosstalk between these two pathways. Downstream of FOXO3 activation, Bim/Bcl2l11 expression is increased and BIM-mediated apoptosis ensues. We propose that miR-155 deletion in LAT-KI mice causes a decrease in lymphoproliferative disease based on this increased BIM-mediated apoptosis. To test the importance of these pathways, which both funnel through BIM, we crossed DM mice with BIM-deficient mice. As shown in Fig 8A and S5 Fig, BIM deficiency largely restored lymphoproliferative disease in DM mice, providing evidence for the importance of BIM in lymphoproliferative disease in the LAT-KI background. A summary of the interacting pathways thought to be impacted by miR155 deletion in CD4^+^ T cells is presented in Fig 8B.

## Discussion

LAT-KI mice exhibit a fatal lymphoproliferative disease dependent on CD4^+^ T cells that kills the mice by about 6 months of age [2, 3] and are a model of genetic lymphoproliferative disease. In a study investigating the role of miRNAs in this disease model, we observed that miR-155 was overexpressed in LAT-KI CD4^+^ T cells [8]. Although we do not know the mechanism whereby miR-155 is overexpressed in LAT-KI T cells, a number of transcription factors that control miR-155 expression have been identified and may be hyper-activated in LAT-KI T cells [10]. To test for a potential role of miR-155 in LAT-KI lymphoproliferative disease, we crossed LAT-KI mice to miR-155 null mice yielding DM mice. Here we conclude that miR-155 does indeed regulate LAT-KI lymphoproliferative disease as the disease was dramatically reduced in DM mice. Other pathways to lymphoproliferative disease not dependent on miR-155 exist though, as evidenced by development of lymphoproliferative disease in older DM mice. Furthermore, we used LAT-KI mice as a discovery tool to probe signaling pathways downstream from miR-155. As summarized in Fig 8, we found that SHIP-1/AKT and PAK1/JNK signaling pathways contribute to BIM-mediated apoptosis downstream from miR-155 and that mTOR links the SHIP-1/AKT and PAK1/JNK pathways. We provide further evidence that pathways through BIM are critical for determining the extent of lymphoproliferative disease in LAT-KI mice by showing that BIM deficiency in DM mice restores disease.

Our data suggest that miR-155 affects the balance between proliferation and apoptosis in T cells. Because we hypothesized that the contribution of miR-155 expression to apoptosis was key to controlling lymphoproliferative disease in LAT-KI mice, we extensively studied molecular mechanisms leading to apoptosis in LAT-KI and DM T cells. Levels of apoptosis in DM compared to LAT-KI T cells were higher as measured by increased AnnexinV staining and levels of active Caspase3, active Caspase9, and PARP. We also found elevated levels of the pro-apoptotic factor BIM in DM compared to LAT-KI T cells. There is ample evidence for the pro-apoptotic functions of BIM [27, 28, 48] and it has been reported that FOXO3 directly induces the transcription of BIM/Bcl2l11 [14, 30–32]. Because the transcription factor FOXO3 is a direct target of miR-155, miR-155 can directly regulate its expression and thus that of BIM [16, 17]. FOXO3, BIM, and phospho-BIM levels were higher in DM than in LAT-KI T cells. In addition to direct regulation, we defined several linked pathways downstream of miR-155 in T cells that converge to regulate the nuclear translocation and activation of FOXO3 (Fig 8). Recently it has been suggested that FOXO3 could play an indirect role in BIM mRNA regulation possibly via other transcription factors or miRNAs [49]. Regardless of the mechanism, we propose that differing levels of FOXO3-initiated, BIM-mediated apoptosis lead to different amounts of lymphoproliferative disease in LAT-KI mice.

We found that two pathways contribute to FOXO3 activity in CD4^+^ T cells, a PAK1/JNK pathway and a SHIP-1/AKT pathway. PAK1 activation of JNK has been shown in numerous cell types [50–54]. PAK1 activation of JNK in Jurkat T cells following TCR cross-linking has been disputed [55]. Our study provides multiple lines of evidence that PAK1 activation increases JNK activation in primary T cells and in Jurkat T cells (Figs 6 and 7). We propose that PAK1 is involved in apoptosis in T cells via the cascade PAK1→JNK→FOXO3→BIM→active Caspase 9 and cytochrome C release.

In addition to this effect of PAK1 on JNK activation, we previously described a role for a PAK1/PLCγ-1/Bam32 complex in ERK activation in both normal CD4^+^ T cells [7] and in CD4^+^ T cells isolated from LAT-KI mice [5]. Furthermore, genetic studies from both our laboratory [5, 6] and others [56] have shown a definitive role for ERK signaling in LAT-KI lymphoproliferative disease. These and other studies have revealed that there are multiple roads leading to ERK activation depending on the signaling environment [57]. This study provides the first evidence that miR-155 modulates ERK activation. Paradoxically, miR-155 deletion delayed LAT-KI lymphoproliferative disease despite causing an increase in ERK activation. This effect is in contrast to other null mutants crossed to LAT-KI mice (RASGRP1 and BAM32), which decreased ERK activation and decreased lymphoproliferative disease [5, 6]. We propose that PAK1/JNK pathway-mediated apoptosis contributes more to the effect on lymphoproliferative disease than PAK1/ERK-mediated proliferation in DM T cells. Both PAK1 and JNK have been associated with cell death [58–60]. Here we propose that miR-155, PAK1 and JNK are part of a common pathway to apoptosis in T cells. Incorporation of a single molecule into distinct pathways of proliferation and apoptosis could provide an elegant way of maintaining tissue homeostasis and preventing carcinogenesis. PAK1 could provide such a function by mediating proliferation through PAK1/ERK action or apoptosis through PAK1/JNK action.

A pro-apoptotic function of PAK1 was described 15 years ago, but no mechanism has been proposed to our knowledge to account for the effect. PAK1 expression is toxic in yeast [59] and its activation kills HeLa cells via an unexplained pathway involving JNK [60]. Here we describe a pathway from PAK1 to apoptosis through JNK activation, however reports on the effect of PAK1 on apoptosis in mammalian cells are mixed. We speculate that the predominant role of PAK1 on survival vs. apoptosis may depend on miR-155 levels. More specifically, we speculate that PAK1 activation is more likely to lead to survival in the presence of high levels of miR-155. In cells exhibiting high miR-155 levels, the strong pro-survival PAK1/ERK pathway (as well as other pathways leading to ERK activation) could dominate the depressed PAK1/JNK apoptotic pathway and miR-155 could act as an oncomir. For example, the breast cancer cell line MDA-MB-175 has high miR-155 expression [61], PAK1 genomic amplification and PAK1 mRNA overexpression. PAK1 siRNA treatment induced apoptosis as measured by AnnexinV staining [62]. Therefore, in this setting, high PAK1 expression promotes survival and decreasing PAK1 expression tips the balance toward apoptosis. On the other hand, HeLa cells and Jurkat T cells do not express miR-155 [17, 63]. Overexpression of active PAK1 accelerated procarcinogen-induced cell death in HeLa cells [60] and overexpression of PAK1 in Jurkat T cells increased expression of BIM and active Caspase 9 (Fig 6). In these contexts (low miR155 expression), PAK1 overexpression could favor the PAK1/JNK pathway and apoptosis. In our system, LAT-KI T cells expressing high miR-155 levels favor a PAK1/ERK/proliferation pathway and DM T cells that do not express miR-155 favor a PAK1/JNK/apoptosis pathway.

The second pathway contributing to FOXO3 activity in CD4^+^ T cells is the SHIP-1/AKT pathway. AKT can be negatively regulated by the action of SHIP-1, a direct target of miR-155, and by phosphorylation by mTORC2 and PDK1. AKT phosphorylation of FOXO3 negatively regulates its activity as it leads to cytoplasmic retention of FOXO3 [39]. High SHIP-1 expression in LAT-KI CD4^+^ T cells (relative to WT) is predicted to lead to depletion of PIP3 at the membrane and less AKT and PDK1 activity because of lack of membrane recruitment via their PH domains [38, 64]. In fact, we show that phosphorylation of S241 of PDK1 (a marker of activation of PDK1) is lower in cells with higher SHIP-1 levels. In DM CD4^+^ T cells, SHIP-1 is expressed at even higher levels than in LAT-KI CD4^+^ T cells, leading to even less AKT and PDK1 activation of mTOR, enhanced PAK1/JNK activity, and more BIM-mediated apoptosis. Although a large body of evidence demonstrates that AKT activates mTOR in numerous cell types [38], Finlay et al. have shown that mTOR can also be regulated by PDK1 (and not AKT) in CD8^+^ T cells [20]. This issue has not been studied in CD4^+^ T cells, but regulation of mTOR by PDK1 rather than by AKT in LAT-KI CD4^+^ T cells would have a similar effect on mTOR. We found a connection between mTOR and PAK1 levels and PAK1 phosphorylation. Low mTOR activity, as seen in DM T cells and as mimicked by Rapamycin, Rapalog, KU, and Raptor siRNA treatment, increased PAK1 signaling whereas mTOR activation, by treatment with leucine, non-essential amino acids and pyruvate, decreased Pak1 signaling. Another finding of our study is the connection of the SHIP1/AKT/PDK1 and Pak1/JNK pathways by mTOR suggested by the negative effect of mTOR on PAK1 protein levels and activity.

miR-155 overexpression has been correlated with various types of cancers, including lymphomas [10, 11]. Forced overexpression of miR-155 in B cells in mice led to the development of B cell lymphomas [65]. In contrast, nanoparticle delivery of anti-miR-155 nucleic acids led to the regression of the lymphomas in those mice. Therefore in those studies and in our study, depletion of miR-155 levels resulted in less disease, i.e. lymphoma or lymphoproliferative disease, as defined by regression or prevention of disease, respectively. However, in the study of Babar et al. just mentioned above, the disease being “cured” was initiated solely by miR-155 overexpression whereas LAT-KI disease results from LAT mutation and downstream miR-155 overexpression, presumably in addition to many other alterations in gene expression. Collectively, these studies point to a critical role for miR-155 in some lymphoproliferative diseases and lymphomas.

Our study also speaks to the importance of combination therapies for cancer treatment. If multiple signaling pathways contribute to cancer cell survival and proliferation, combination chemotherapy will be more effective than single agent chemotherapy. There is evidence in human and mouse tumor cells [66, 67] that mTOR inhibition activates ERK phosphorylation. In addition, MEK inhibitor treatment of hormone-refractory human prostate cancer cells induces mTOR phosphorylation [68]. Because of the evidence that these two pathways (MEK/ERK and PI3K/mTOR) can cross-activate, combination therapies have been tried and found to be effective. Combination mTOR/MEK inhibitor treatment resulted in decreased tumor size in MCF-7 human breast cancer xenografts [66], decreased androgen-dependent prostate cancer cell growth in vitro [68], and decreased androgen-independent prostate cancer cell growth in vitro and in vivo [69]. The latter study also showed that the decrease in prostate cancer cell growth was through upregulation of BIM. Our results provide additional rationale for combination mTOR/MEK inhibitor therapy because in addition to the anti-proliferative effects of MEK inhibitor treatment, mTOR inhibition resulting in enhanced PAK1/JNK/FOXO3-mediated apoptosis could inhibit tumor progression.

Within the PI3K/mTOR pathway, combination PI3K and mTOR inhibitors have been used for the treatment of lymphomas. Interestingly, resistance to selective PI3K inhibitors and dual PI3K/mTOR inhibitors was mediated by PAK1 [70]. These results are consistent with our work showing that PI3K/mTOR inhibitors increase PAK1 signaling. Walsh et al. propose combining PI3K/mTOR inhibitors with PAK1 inhibitors. However, our results emphasize that PI3K/mTOR inhibition increases PAK1-mediated BIM upregulation (via FOXO3 and BIM phosphorylation by JNK) as well as PAK1-mediated ERK activation. Instead of global PAK1 inhibition, we suggest selective MEK/ERK pathway inhibition for combination with PI3K/mTOR inhibition.

We have used the LAT-KI model as a discovery tool to uncover signaling pathways that are important in lymphoproliferative disease and perhaps in lymphomagenesis as well. We found that miR-155 is elevated in LAT-KI T cells as well as it is in many cancers and especially lymphomas. Downregulation of miR-155 in cancer patients may become possible with future technological advances. However, it is important to bear in mind that loss of miR155 leads to a decrease in tumor-fighting ability by CD8^+^ T cells, macrophages, and dendritic cells [71–73]. In this light, therapies targeted to specific cancer cells should be considered. We also uncovered new signaling pathways that mediate miR-155 regulation in T cells and cross talk between them. Understanding these signaling pathways may provide new information relevant to designing inhibitor combinations to slow uncontrolled growth of monoclonal and polyclonal lymphoid populations.

## Supporting Information

S1 Fig miR-155 deficiency delays Th2 lymphoproliferative disease. **A.** Photomicrographs of H&E-stained sections of lungs from mice of the indicated genotypes (10X objective) show reduced lymphocyte infiltration in lungs of DM compared to LAT-KI mice. Ages of the mice were 11.5 wks (WT), 34 wks (miR155^-/-^), 11.5 wks (LAT-KI) and 11 wks (DM). Results are representative of 6 experiments. **B.** Intracellular cytokine production by lymph node CD4^+^ T cells from mice of the indicated genotypes. The ages of the mice were 10 wks (WT), 9 wks (miR155^-/-^), 9 wks (LAT-KI), 16 wks (LAT-KI-old), 9 wks (DM), and 20 wks (DM-old). Results are representative of 2 experiments.
(PDF)

S2 Fig miR-155 levels regulate PAK1 and downstream JNK activity. **A.** Jurkat T cells were transfected with specific PAK1 siRNA pool or control siRNAs (200 nM). After 24h, cells were transfected with YFP (used as a negative control) or BAM32-YFP cDNAs (10 **μ**g). After an additional 20h, cells were stimulated with **α**CD3**ε** (2 **μ**g/ml) for 0, 3 or 10 min. Then SDS WCLs were prepared and analyzed by WB (n = 3). **B.** Negatively selected CD4^+^ T cells from the indicated genotypes were rested for 6h then stimulated with low dose **α**CD3**ε**/CD4 (5 **μ**g/ml) for 0, 3, or 10 min. Ages of the mice were 11 wks (WT), 12 wks (BAM32^-/-^ and LAT-KI), and 14 wks (LAT-BAM). **C.** miR-155 was overexpressed in mouse CD4^+^ T cells by retroviral infection. Mock infection was performed as a negative control. In both cases, GFP was expressed to identify infected cells. Sorted GFP^+^ CD4^+^ T cells were stimulated with **α**CD3**ε**/CD4 (10 **μ**g/ml) for 0, 3 or 10 min. SDS WCLs were analyzed by WB (n = 2). **D.** Verification of MEK and JNK inhibitor efficiency. Before cell fractionation was performed to study PAK1/JNK-mediated FOXO3 nuclear import in Fig 6B, aliquots of Flag-PAK1 transfected cells left untreated (- inhibitor) or incubated with one of the two inhibitors (+ inhibitor) were used to make WCLs that were analyzed by WB (n = 3). MEK and JNK expression were determined on separate gels from pMEK and pJNK expression because of the inability of pMEK and total JNK Abs to be properly stripped.
(PDF)

S3 Fig PLC-1γ/PAK1 cooperation enhances BIM-mediated apoptosis. **A.** Jurkat T cells were transfected either with PLC-**γ**1^CI^-HA, Flag-PAK1, or both cDNAs (10 **μ**g each). 48h post-transfection, cytosolic fractions were examined for cytochrome C levels by WB (n = 4). **B.** Jurkat T cells were transfected either with PLC-**γ**1^CI^-HA, Flag-PAK1, or both cDNAs (10 **μ**g each). Caspase 9 inhibitor (z-LEHD-fmk, 100 **μ**M) was added 4h after transfection to minimize drug toxicity. 40h post-transfection, cells were lysed. Lysates (75%) were subjected to an active Caspase 9 IP and the 25% remaining lysates were used to prepare WCLs. Samples were then analyzed by WB (n = 3).
(PDF)

S4 Fig mTOR inhibition by Rapalogs and nutrients alters PAK1 signaling. A. mTOR inhibition by Rapalogs increases PAK1 signaling. Jurkat T cells were treated with Deforolimus, Everolimus, or Temsirolimus (100 nM) for 0, 2, 4, or 6h. SDS WCLs were prepared then analyzed by WB (n = 2). **B.** To measure PAK1 stability, Jurkat T cells were starved (0.5% FCS) for 16h then pre-treated for 2h with cycloheximide (CHX, 50 **μ**g/ml). After CHX pre-treatment, cells were not washed and Rapamycin (100 nM) was added to the media. Every hour SDS WCLs were made. Quantitation of the WB (n = 3) can be found in Fig 7E. **C.** mTOR activation by nutrients decreases PAK1 levels and PAK1-controlled BIM levels. Jurkat T cells were incubated in RPMI 1640 supplemented either with L-Leucine (2.5 or 5 mM), sodium pyruvate, or non-essential amino acids (AAs) at 1X levels as suggested by the manufacturer. SDS WCLs were prepared then analyzed by WB (n = 5). **D.** Jurkat T cells were transfected with PAK1 or control siRNAs (200 **μ**M). 48h post-transfection, cells were treated with Rapamycin (100 nM) combined with either MEK inhibitor (U0126, 20 **μ**M) or low dose JNK inhibitor (SP600125, 10 **μ**M) for 16h and lysed. Lysates (75%) were subjected to an active Caspase 9 IP and the 25% remaining lysates were used to make WCLs. Samples were analyzed by WB (n = 3). The first two lanes (JE6.1 and JE6.1+etoposide) are negative IgG IP controls. **E.** Verification of MEK and JNK inhibitor efficiency by WB using WCL aliquots obtained from S4 Fig (D, n = 3).
(PDF)

S5 Fig BIM deficiency increases lymphoproliferative disease in LAT-KI x miR-155^-/-^ mice. CD4 and CD8 surface marker expression as measured by flow cytometry. Ages of the mice were 7 wks. The results are representative of 6 experiments.
(PDF)

S1 Text Supplementary Materials and Methods. (PDF)
