# Supplementary material for: miR-155 Controls Lymphoproliferation in LAT Mutant Mice by Restraining T-Cell Apoptosis via SHIP-1/mTOR and PAK1/FOXO3/BIM Pathways
Source: PLoS One. 2015 Jun 29;10(6):e0131823. doi: 10.1371/journal.pone.0131823 (PMC4487994; doi:10.1371/journal.pone.0131823)
Supplement: S1 Fig — A. Photomicrographs of H&E-stained sections of lungs from mice of the indicated genotypes (10X objective) show reduced lymphocyte infiltration in lungs of DM compared to LAT-KI mice. Ages of the mice were 11.5 wks (WT), 34 wks (miR155-/-), 11.5 wks (LAT-KI) and 11 wks (DM). Results are representative of 6 experiments. B. Intracellular cytokine production by lymph node CD4+ T cells from mice of the indicated genotypes. The ages of the mice were 10 wks (WT), 9 wks (miR155-/-), 9 wks (LAT-KI), 16 wks (LAT-KI-old), 9 wks (DM), and 20 wks (DM-old). Results are representative of 2 experiments. (PDF) [file pone.0131823.s001.pdf]

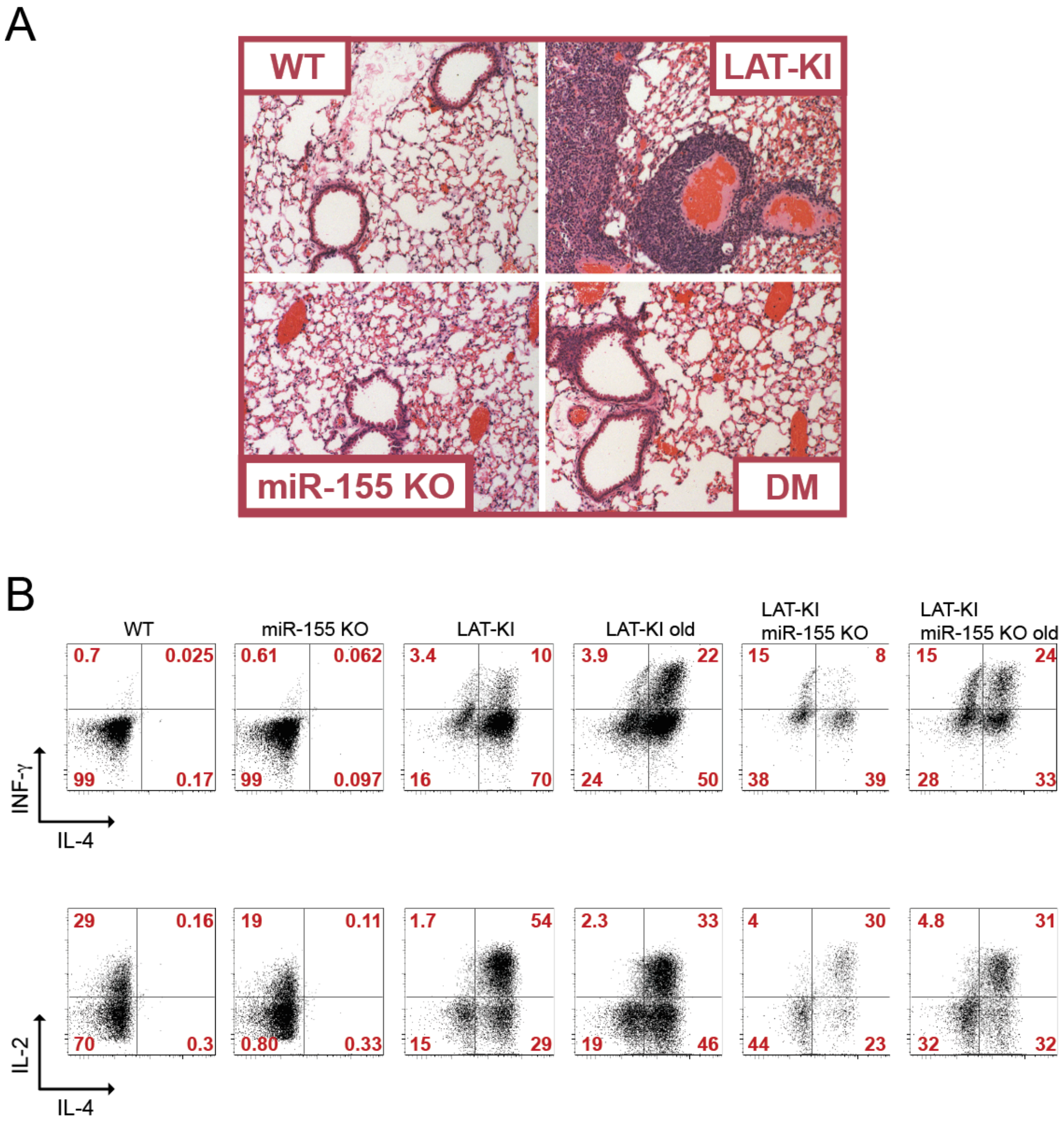


**S1 Fig. miR-155 deficiency delays Th2 lymphoproliferative disease.**

**A.** Photomicrographs of H&E-stained sections of lungs from mice of the indicated genotypes (10X objective) show reduced lymphocyte infiltration in lungs of DM compared to LAT-KI mice. Ages of the mice were 11.5 wks (WT), 34 wks (miR155$^{-/-}$), 11.5 wks (LAT-KI) and 11 wks (DM). Results are representative of 6 experiments. **B.** Intracellular cytokine production by lymph node CD4$^{+}$ T cells from mice of the indicated genotypes. The ages of the mice were 10 wks (WT), 9 wks (miR155$^{-/-}$), 9 wks (LAT-KI), 16 wks (LAT-KI-old), 9 wks (DM), and 20 wks (DM-old). Results are representative of 2 experiments.
