# Supplementary material for: miR-155 Controls Lymphoproliferation in LAT Mutant Mice by Restraining T-Cell Apoptosis via SHIP-1/mTOR and PAK1/FOXO3/BIM Pathways
Source: PLoS One. 2015 Jun 29;10(6):e0131823. doi: 10.1371/journal.pone.0131823 (PMC4487994; doi:10.1371/journal.pone.0131823)
Supplement: S2 Fig — A. Jurkat T cells were transfected with specific PAK1 siRNA pool or control siRNAs (200 nM). After 24h, cells were transfected with YFP (used as a negative control) or BAM32-YFP cDNAs (10 μg). After an additional 20h, cells were stimulated with αCD3ε (2 μg/ml) for 0, 3 or 10 min. Then SDS WCLs were prepared and analyzed by WB (n = 3). B. Negatively selected CD4+ T cells from the indicated genotypes were rested for 6h then stimulated with low dose αCD3ε/CD4 (5 μg/ml) for 0, 3, or 10 min. Ages of the mice were 11 wks (WT), 12 wks (BAM32-/- and LAT-KI), and 14 wks (LAT-BAM). C. miR-155 was overexpressed in mouse CD4+ T cells by retroviral infection. Mock infection was performed as a negative control. In both cases, GFP was expressed to identify infected cells. Sorted GFP+ CD4+ T cells were stimulated with αCD3ε/CD4 (10 μg/ml) for 0, 3 or 10 min. SDS WCLs were analyzed by WB (n = 2). D. Verification of MEK and JNK inhibitor efficiency. Before cell fractionation was performed to study PAK1/JNK-mediated FOXO3 nuclear import in Fig 6B, aliquots of Flag-PAK1 transfected cells left untreated (- inhibitor) or incubated with one of the two inhibitors (+ inhibitor) were used to make WCLs that were analyzed by WB (n = 3). MEK and JNK expression were determined on separate gels from pMEK and pJNK expression because of the inability of pMEK and total JNK Abs to be properly stripped. (PDF) [file pone.0131823.s002.pdf]

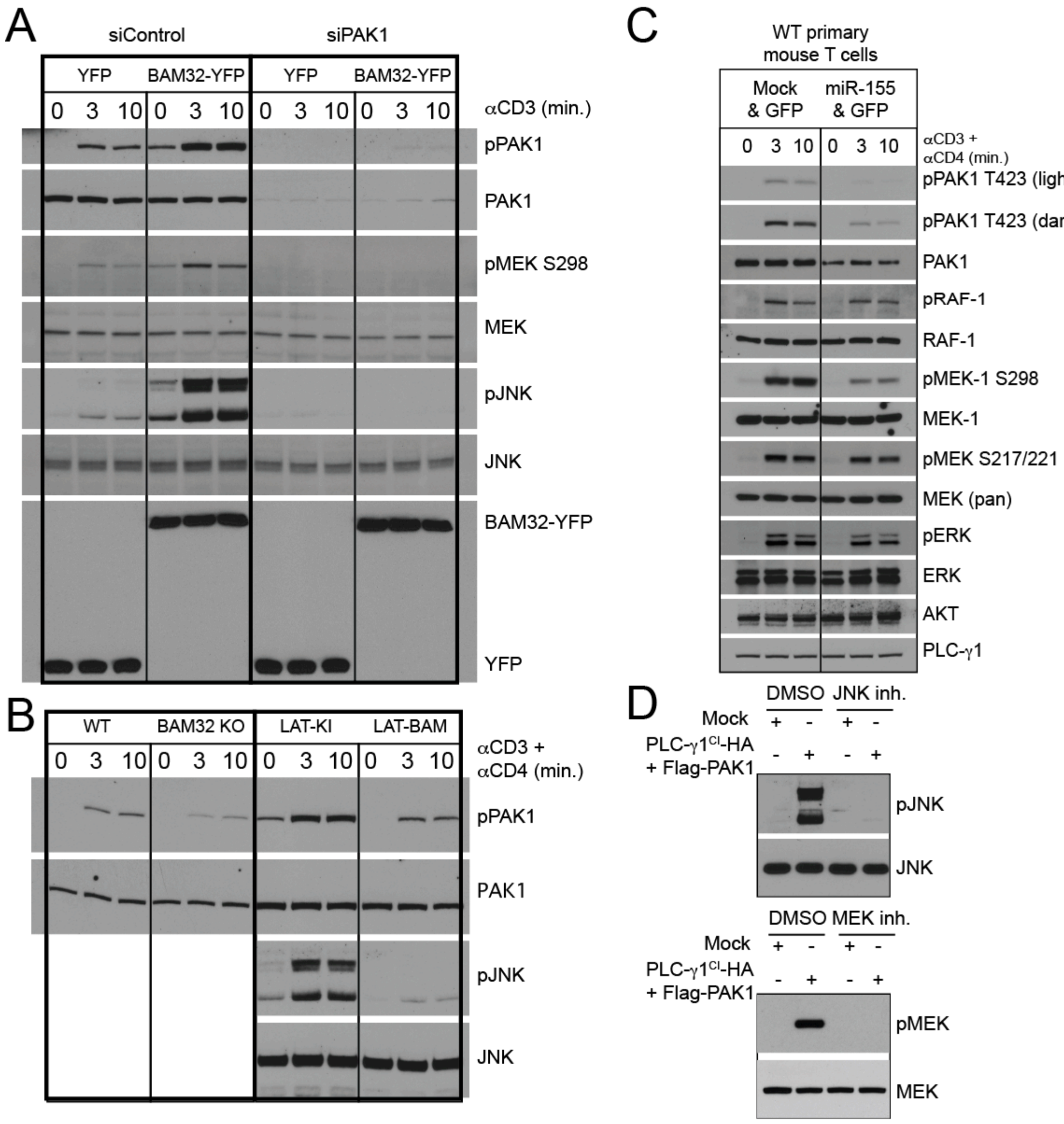

S2 Fig. miR-155 levels regulate PAK1 and downstream JNK activity.

A. Jurkat T cells were transfected with specific PAK1 siRNA pool or control siRNAs (200 nM). After 24h, cells were transfected with YFP (used as a negative control) or BAM32-YFP cDNAs (10 μg). After an additional 20h, cells were stimulated with αCD3ε (2 μg/ml) for 0, 3 or 10 min. Then SDS WCLs were prepared and analyzed by WB (n=3). **B.** Negatively selected $CD4^+$ T cells from the indicated genotypes were rested for 6 hours then stimulated with low dose αCD3ε/CD4 (5 μg/ml) for 0, 3, or 10 minutes. Ages of the mice were 11 wks (WT), 12 wks ($BAM32^{-/-}$ and LAT-KI), and 14 wks (LAT-BAM). **C.** miR-155 was overexpressed in mouse $CD4^+$ T cells by retroviral infection. Mock infection was performed as a negative control. In both cases, GFP was expressed to identify infected cells. Sorted GFP^+ $CD4^+$ T cells were stimulated with αCD3ε/CD4 (10 μg/ml) for 0, 3 or 10 min. SDS WCLs were analyzed by WB (n=2). **D.** Verification of MEK and JNK inhibitor efficiency. Before cell fractionation was performed to study PAK1/JNK-mediated FOXO3 nuclear import in Fig. 6B, aliquots of Flag-PAK1 transfected cells left untreated (- inhibitor) or incubated with one of the two inhibitors (+ inhibitor) were used to make WCLs that were analyzed by WB (n=3). MEK and JNK expression were determined on separate gels from pMEK and pJNK expression because of the inability of pMEK and total JNK Abs to be properly stripped.
