# Supplementary material for: miR-155 Controls Lymphoproliferation in LAT Mutant Mice by Restraining T-Cell Apoptosis via SHIP-1/mTOR and PAK1/FOXO3/BIM Pathways
Source: PLoS One. 2015 Jun 29;10(6):e0131823. doi: 10.1371/journal.pone.0131823 (PMC4487994; doi:10.1371/journal.pone.0131823)
Supplement: S3 Fig — A. Jurkat T cells were transfected either with PLC-γ1CI-HA, Flag-PAK1, or both cDNAs (10 μg each). 48h post-transfection, cytosolic fractions were examined for cytochrome C levels by WB (n = 4). B. Jurkat T cells were transfected either with PLC-γ1CI-HA, Flag-PAK1, or both cDNAs (10 μg each). Caspase 9 inhibitor (z-LEHD-fmk, 100 μM) was added 4h after transfection to minimize drug toxicity. 40h post-transfection, cells were lysed. Lysates (75%) were subjected to an active Caspase 9 IP and the 25% remaining lysates were used to prepare WCLs. Samples were then analyzed by WB (n = 3). (PDF) [file pone.0131823.s003.pdf]

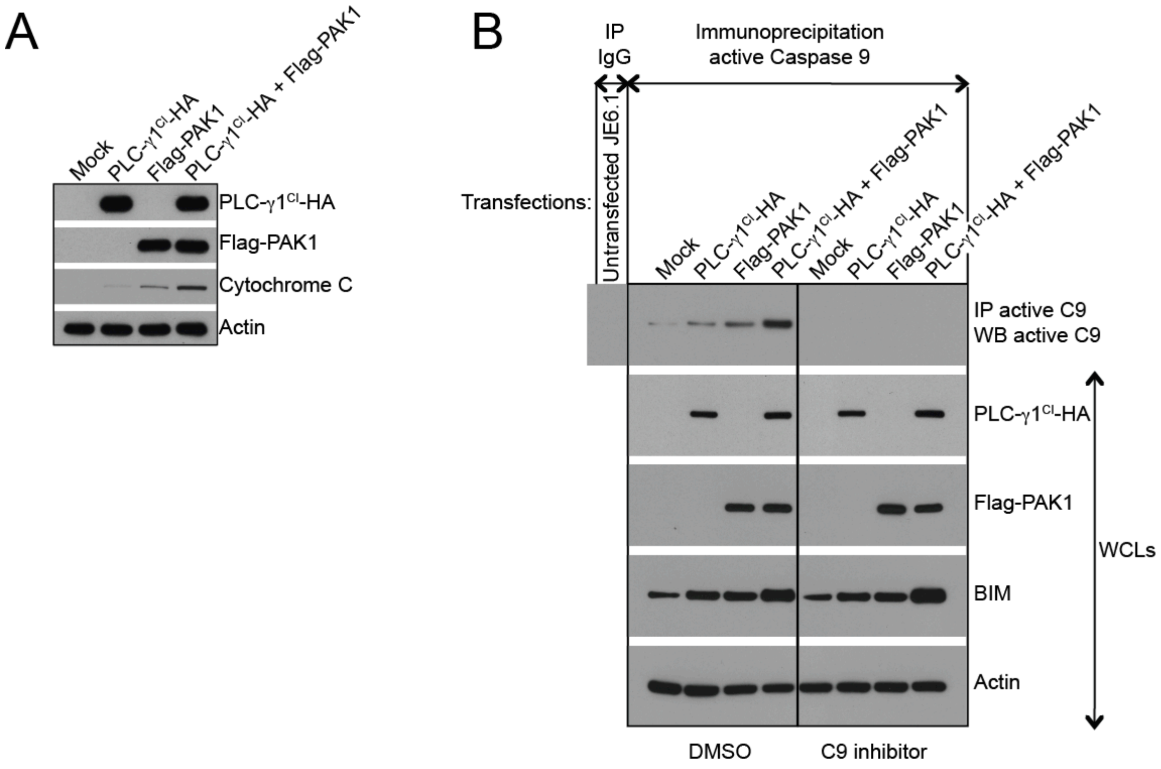

S3 Fig. PLC-γ1/PAK1 cooperation enhances BIM-mediated apoptosis.

A. Jurkat T cells were transfected either with PLC-γ1^{CI}-HA, Flag-PAK1, or both cDNAs (10 µg each). 48h post-transfection, cytosolic fractions were examined for cytochrome C levels by WB (n=4). **B.** Jurkat T cells were transfected either with PLC-γ1^{CI}-HA, Flag-PAK1, or both cDNAs (10 µg each). Caspase 9 inhibitor (z-LEHD-fmk, 100 µM) was added 4h after transfection to minimize drug toxicity. 40h post-transfection, cells were lysed. Lysates (75%) were subjected to an active Caspase 9 IP and the 25% remaining lysates were used to prepare WCLs. Samples were then analyzed by WB (n=3).
