# Supplementary material for: miR-155 Controls Lymphoproliferation in LAT Mutant Mice by Restraining T-Cell Apoptosis via SHIP-1/mTOR and PAK1/FOXO3/BIM Pathways
Source: PLoS One. 2015 Jun 29;10(6):e0131823. doi: 10.1371/journal.pone.0131823 (PMC4487994; doi:10.1371/journal.pone.0131823)
Supplement: S4 Fig — A. mTOR inhibition by Rapalogs increases PAK1 signaling. Jurkat T cells were treated with Deforolimus, Everolimus, or Temsirolimus (100 nM) for 0, 2, 4, or 6h. SDS WCLs were prepared then analyzed by WB (n = 2). B. To measure PAK1 stability, Jurkat T cells were starved (0.5% FCS) for 16h then pre-treated for 2h with cycloheximide (CHX, 50 μg/ml). After CHX pre-treatment, cells were not washed and Rapamycin (100 nM) was added to the media. Every hour SDS WCLs were made. Quantitation of the WB (n = 3) can be found in Fig 7E. C. mTOR activation by nutrients decreases PAK1 levels and PAK1-controlled BIM levels. Jurkat T cells were incubated in RPMI 1640 supplemented either with L-Leucine (2.5 or 5 mM), sodium pyruvate, or non-essential amino acids (AAs) at 1X levels as suggested by the manufacturer. SDS WCLs were prepared then analyzed by WB (n = 5). D. Jurkat T cells were transfected with PAK1 or control siRNAs (200 μM). 48h post-transfection, cells were treated with Rapamycin (100 nM) combined with either MEK inhibitor (U0126, 20 μM) or low dose JNK inhibitor (SP600125, 10 μM) for 16h and lysed. Lysates (75%) were subjected to an active Caspase 9 IP and the 25% remaining lysates were used to make WCLs. Samples were analyzed by WB (n = 3). The first two lanes (JE6.1 and JE6.1+etoposide) are negative IgG IP controls. E. Verification of MEK and JNK inhibitor efficiency by WB using WCL aliquots obtained from S4 Fig (D, n = 3). (PDF) [file pone.0131823.s004.pdf]

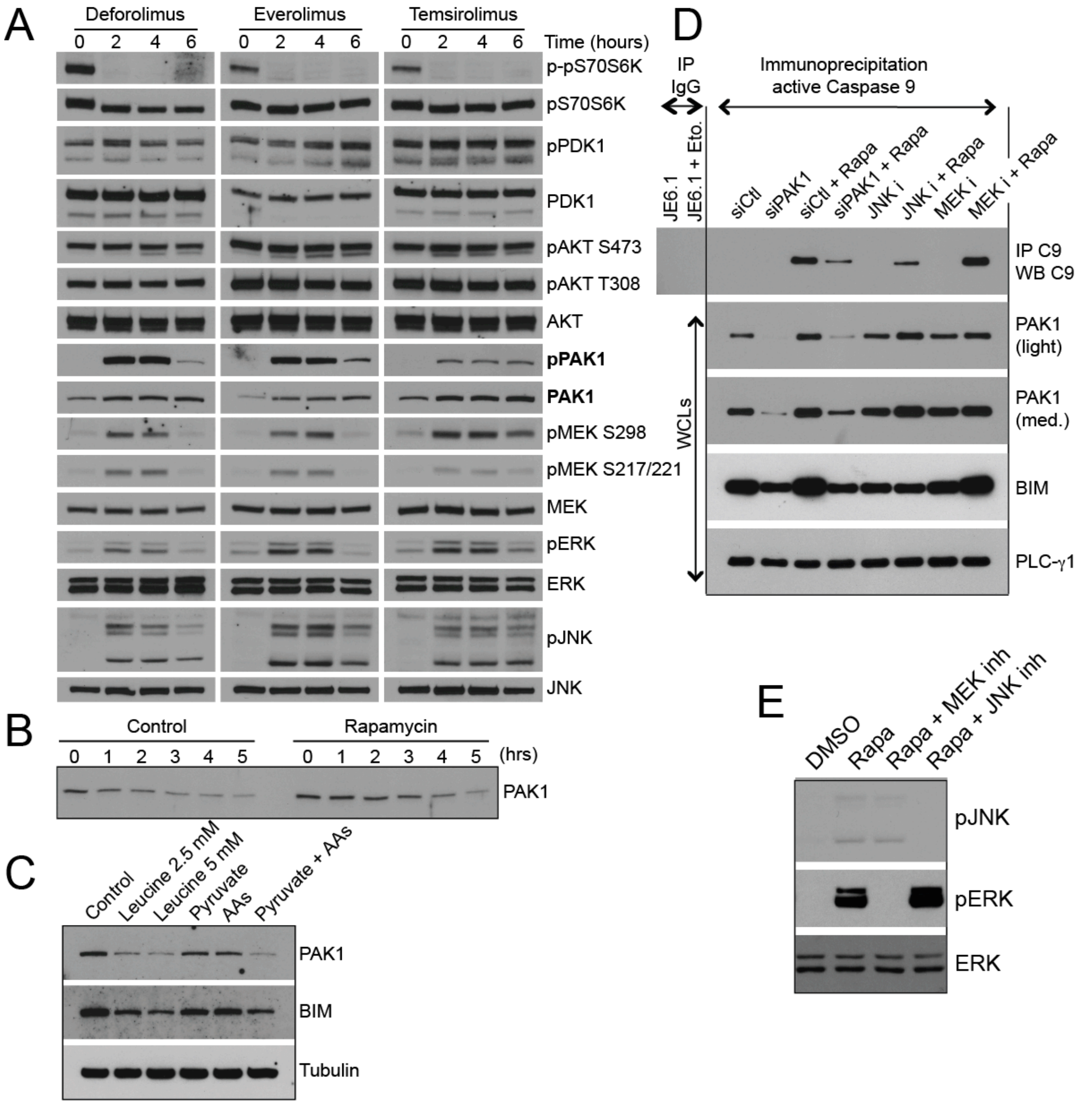

S4 Fig. mTOR inhibition by Rapalogs and nutrients alters PAK1 signaling.

A. mTOR inhibition by Rapalogs increases PAK1 signaling. Jurkat T cells were treated with Deforolimus, Everolimus, or Temsirolimus (100 nM) for 0, 2, 4, or 6h. SDS WCLs were prepared then analyzed by WB (n=2). **B.** To measure PAK1 stability, Jurkat T cells were starved (0.5% FCS) for 16h then pre-treated for 2h with cycloheximide (CHX, 50 μg/ml). After CHX pre-treatment, cells were not washed and Rapamycin (100 nM) was added to the media. Every hour SDS WCLs were made. Quantitation of the WB (n=3) can be found in Fig. 7E. **C.** mTOR activation by nutrients decreases PAK1 levels and PAK1-controlled BIM levels. Jurkat T cells were incubated in RPMI 1640 supplemented either with L-Leucine (2.5 or 5 mM), sodium pyruvate, or non-essential amino acids (AAs) at 1X levels as suggested by the manufacturer. SDS WCLs were prepared then analyzed by WB (n=5). **D.** Jurkat T cells were transfected with PAK1 or control siRNAs (200 μM). 48h post-transfection, cells were treated with Rapamycin (100 nM) combined with either MEK inhibitor (U0126, 20 μM) or low dose JNK inhibitor (SP600125, 10 μM) for 16 hours and lysed. Lysates (75%) were subjected to an active Caspase 9 IP and the 25% remaining lysates were used to make WCLs. Samples were analyzed by WB (n=3). The first two lanes (JE6.1 and JE6.1+etoposide) are negative IgG IP controls. **E.** Verification of MEK and JNK inhibitor efficiency by WB using WCL aliquots obtained from Fig. S4D (n=3).
