# Supplementary material for: miR-155 Controls Lymphoproliferation in LAT Mutant Mice by Restraining T-Cell Apoptosis via SHIP-1/mTOR and PAK1/FOXO3/BIM Pathways
Source: PLoS One. 2015 Jun 29;10(6):e0131823. doi: 10.1371/journal.pone.0131823 (PMC4487994; doi:10.1371/journal.pone.0131823)
Supplement: S5 Fig — CD4 and CD8 surface marker expression as measured by flow cytometry. Ages of the mice were 7 wks. The results are representative of 6 experiments. (PDF) [file pone.0131823.s005.pdf]

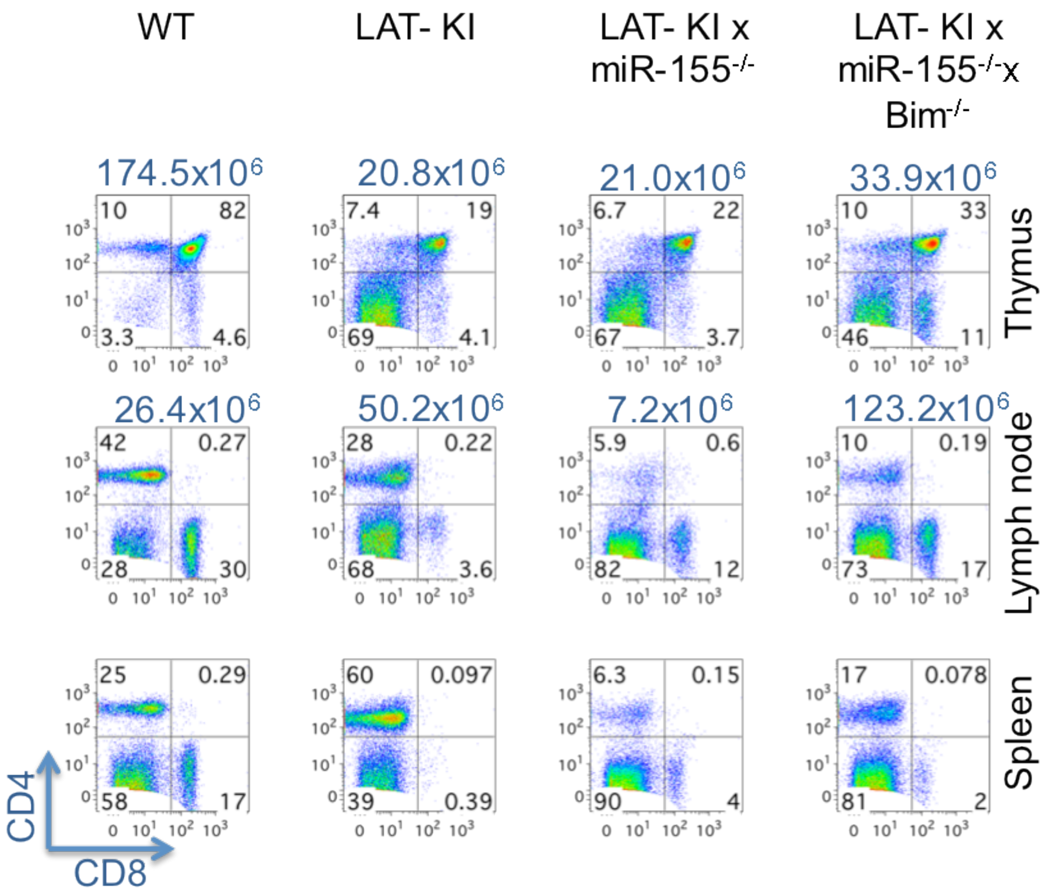

S5 Fig. BIM deficiency increases lymphoproliferative disease in LAT-KI x miR-155$^{-/-}$ mice. CD4 and CD8 surface marker expression as measured by flow cytometry. Ages of the mice were 7 wks. The results are representative of 6 experiments.
