# Supplementary material for: miR-155 Controls Lymphoproliferation in LAT Mutant Mice by Restraining T-Cell Apoptosis via SHIP-1/mTOR and PAK1/FOXO3/BIM Pathways
Source: PLoS One. 2015 Jun 29;10(6):e0131823. doi: 10.1371/journal.pone.0131823 (PMC4487994; doi:10.1371/journal.pone.0131823)
Supplement: S1 Text — (PDF) [file pone.0131823.s006.pdf]

S1 Text. Supplementary Materials and Methods

Antibodies for western-blotting. PI3K/mTOR pathway: Anti-SHIP-1 (P1C1) mouse mAb (sc-8425) was from Santa Cruz Biotechnology. Anti-pSHIP-1 Y1020 rabbit Ab (3941), anti-pPI3K p85 Y458)/p55 Y199 rabbit Ab (4228), anti-PI3K p85 (19H8) rabbit mAb (4257), anti-pAKT S473 (D9E XP) rabbit mAb (4060), anti-pAKT T308 (C31E5E) rabbit mAb (2965), anti-pan AKT (11E7) rabbit mAb (4685), anti-pPDK1 S241 (C49H2) rabbit mAb (3438), anti-PDK1 (D37A7) rabbit mAb (5662), anti-p-p70 S6K T389 (108D2) rabbit mAb (9234), and anti-p70 S6K (49D7) rabbit mAb (2708) were all from Cell Signaling Technology. MAPK pathway: Anti-PAK1 (H-300) rabbit Ab (sc-11394) was from Santa Cruz Biotechnology. Anti-PAK1 rabbit Ab (2602), anti-pPAK1 S199/204/Pak2 S192/197 rabbit Ab (2605), anti-pPAK1 T423/PAK2 T402 rabbit Ab (2601), anti-pJNK/SAPK T183/Y185 rabbit Ab (9251), anti-JNK/SAPK (56G8) rabbit mAb (9258), anti-p-RAF-1/c-RAF S338 (56A6) rabbit mAb, anti-RAF-1/c-RAF rabbit Ab (9422), anti-pMEK-1 S298 rabbit Ab (9128), anti-pMEK-1/2 S217/221 rabbit Ab (9121), anti-MEK-1 rabbit Ab (9124), anti-MEK-1/2 (L38C12) mouse mAb (4694), anti-pERK-1/2 (p44/42 MAPK) T202/TY204 rabbit mAb (D13.14.4E XP), anti-ERK-1/2 (p44/42 MAPK) rabbit Ab (9102), anti-p-p38 T180/Y182 rabbit mAb (9215), and anti-p38 rabbit Ab (9212) were all from Cell Signaling Technology. Apoptosis: Anti-pBIM (S65, Jnk site) rabbit Ab was from Millipore. Anti-BIM (C34C5) rabbit mAb (2933), anti-BNIP3 (rodent specific) rabbit Ab (3769), anti-Full-length Caspase 3 (8G10, mouse & human) rabbit mAb (9665) and anti-Cleaved Caspase 3 (Asp175, 5A1E, mouse & human) rabbit mAb (9664) were from Cell Signaling Technology. Anti-Full-length Caspase 9 p35 (H-170, mouse & human) rabbit Ab (sc-8355) was from Santa Cruz Biotechnology. Anti-Cleaved Caspase 9 (Asp353, mouse specific) rabbit Ab (9509), anti-Cleaved Caspase 9 (Asp315, human specific) rabbit Ab (9505), anti-Full-length PARP (46D11, mouse & human) rabbit mAb (9532), anti-Cleaved PARP (Asp214, 7C9, mouse specific) mouse mAb (9548), anti-FOXO3a (D19A7) rabbit mAb (12829), anti-FOXO1 (C29H4) rabbit mAb (2880), and anti-Cytochrome C (D18C7) rabbit mAb (11940) were from Cell Signaling Technology. Miscellaneous antibodies: Anti-pGSK3β S9 (AKT site) rabbit mAb (9336) and anti-GSK3β (27C10) rabbit mAb (9315) were from Cell Signaling Technology. Anti-β-Tubulin (H-235) rabbit Ab (sc-9104), anti-Lamin B (C-20)

goat Ab (sc-62-16), anti-PLC-γ1 (E-12) mouse mAb (sc-7290), donkey anti-goat IgG-HRP (sc-2033) and irrelevant normal rabbit IgG for control immunoprecipitation (sc-2027) were from Santa Cruz Biotechnology. Anti-β-Actin (8H10D10) mouse mAb (3700) was from Cell Signaling Technology. Goat anti-mouse IgG-HRP (12-349) and goat anti-rabbit IgG-HRP (12-348) were both from Millipore.

Drugs and reagents. Caspase 9 inhibitor (Z-LEHD-FMK, C1355), Cycloheximide (CHX, C4859), PI3K inhibitor (LY-294002, L9908) and its control inactive enantiomer (LY-303511, L2786), Jnk inhibitor (SP600125, S5567) were all purchased from Sigma-Aldrich. Mek inhibitor (U0126, 9903) was from Cell Signaling Technology. All mTOR inhibitors were from Selleckchem. All inhibitors were dissolved in DMSO, with a final DMSO concentration of <2.5% vol/vol in RPMI 1640.

Immunoprecipitation (Caspase 9). Jurkat E6.1 cells (20-25x10^6) transiently transfected or not either with the indicated plasmid(s) or siRNAs were then treated or not with the indicated drugs and maintained in the incubator in complete culture media supplemented with 20 mM HEPES for the indicated times. At the end of the experiment, cells were washed and pelleted with RPMI + HEPES (RT). Pelleted cells were then lysed in 600 μl of ice-cold lysis buffer (25 mM Tris-HCl pH 8.0, 150 mM NaCl, 5 mM EDTA, 5 mM EGTA, 10 mM sodium pyrophosphate, 5 mM Na_3VO_4, 10 mM NaF, 1% Brij 97, 0.5% Octyl-β-D-glucoside supplemented with complete protease inhibitor tablets from Roche) for 90 min. on a rotator placed at 4°C. Lysates were clarified at 12 000 g for 20 min at 4°C to remove detergent-insoluble material. Soluble material was pre-cleared with 4 μg of normal rabbit IgG bound to 20 μl of Protein A/G Plus-Agarose beads for 1 h at 4°C. The pre-cleared samples were incubated with rotation for 2 h at 4 °C with 4 μg of anti-cleaved Caspase 9 rabbit Ab which had been previously conjugated to 20 μl Protein A/G Plus-Agarose beads. The immunoprecipitates were washed three times with ice-cold lysis buffer then twice with ice-cold lysis buffer containing only 10% detergent. This step was performed to remove the excess of Brij 97 and Octyl-β-D-glucoside because these non-ionic detergents interfere with SDS-PAGE. The immunoprecipitates were eluted from the

beads by boiling in 50 μl SDS 2.5X sample buffer (60 mM Tris-HCl, pH 6.8, 10% glycerol, 5 mM EDTA, 10% SDS, 10% fresh β-ME, 200 mM fresh DTT, 0.01% bromophenol blue). The reason why such a large amount of reducing agents were added to the sample buffer is because anti-Cleaved Caspase 9 rabbit Ab is hardly fully reduced with standard amount of β-ME (5%); partially reduced IgGs can give fragments that migrate on SDS-PAGE close to the active Caspase 9 band thus preventing clean WBs. Traditional HRP-conjugated secondary Abs detect both native and denatured heavy and light Ab chains. Because they recognize both IP and WB Abs when the latter are from the same species, traditional HRP-conjugated secondary Abs produce high background around 55 and 25 kDa that masks the signals of interest on WBs. In this paper, we used the same rabbit Ab to pull-down active Caspase 9 fragments and to detect them by WB. To ensure the cleanest WBs, we used Conformation Specific mouse anti-rabbit IgG (L27A9) mAb (3678) from Cell Signaling Technology. It was added as a bridging Ab prior to incubation with an anti-mouse IgG HRP linked secondary Ab.

Immunoblotting. For Jurkat T cells, $4x10^5$ cell equivalents were separated by SDS-PAGE using 10% Criterion Precast polyacrylamide gels (Bio-Rad). For pMek-1 S298 WB, the amount of sample was doubled. For mouse experiments, samples were loaded at $0.5x10^6$ cells per lane. The separated proteins were then transferred to polyvinylidene difluoride (PVDF) membranes. Membranes were blocked for 1 h at RT with pre-warmed at 37°C TBST (10 mM Tris (pH 8.0), 150 mM NaCl, and 0.05% Tween 20) with 5% non-fat dry milk and 1% BSA. The membranes were incubated overnight at 4°C with primary Abs diluted in TBST with 5% non-fat dry milk, 1% BSA, followed by a 60 minute incubation at RT with the appropriate secondary Ab-HRP diluted in TBST with 5% milk, 1% BSA (1/20000). All primary Abs from Cell Signaling Technology were used at 1/2000, except for β-Actin and β-Tubulin Abs that were diluted at 1/5000. The WBs (except for β-Actin and β-Tubulin) were then visualized by enhanced chemiluminescence (ECL) using 1 vol of SuperSignal West Pico ECL plus 1/10 vol of SuperSignal West Femto ECL from Pierce. For β-Actin and β-Tubulin WBs, standard detection ECL from Amersham was used instead.
